# Functional Fluorescent Protein Insertions in Herpes Simplex Virus gB Report on gB Conformation before and after Execution of Membrane Fusion

**DOI:** 10.1371/journal.ppat.1004373

**Published:** 2014-09-18

**Authors:** John R. Gallagher, Doina Atanasiu, Wan Ting Saw, Matthew J. Paradisgarten, J. Charles Whitbeck, Roselyn J. Eisenberg, Gary H. Cohen

**Affiliations:** 1 Department of Microbiology, School of Dental Medicine, University of Pennsylvania Philadelphia, Pennsylvania, United States of America; 2 Department of Pathobiology, School of Veterinary Medicine, University of Pennsylvania, Philadelphia, Pennsylvania, United States of America; Northwestern University, United States of America

## Abstract

Entry of herpes simplex virus (HSV) into a target cell requires complex interactions and conformational changes by viral glycoproteins gD, gH/gL, and gB. During viral entry, gB transitions from a prefusion to a postfusion conformation, driving fusion of the viral envelope with the host cell membrane. While the structure of postfusion gB is known, the prefusion conformation of gB remains elusive. As the prefusion conformation of gB is a critical target for neutralizing antibodies, we set out to describe its structure by making genetic insertions of fluorescent proteins (FP) throughout the gB ectodomain. We created gB constructs with FP insertions in each of the three globular domains of gB. Among 21 FP insertion constructs, we found 8 that allowed gB to remain membrane fusion competent. Due to the size of an FP, regions in gB that tolerate FP insertion must be solvent exposed. Two FP insertion mutants were cell-surface expressed but non-functional, while FP insertions located in the crown were not surface expressed. This is the first report of placing a fluorescent protein insertion within a structural domain of a functional viral fusion protein, and our results are consistent with a model of prefusion HSV gB constructed from the prefusion VSV G crystal structure. Additionally, we found that functional FP insertions from two different structural domains could be combined to create a functional form of gB labeled with both CFP and YFP. FRET was measured with this construct, and we found that when co-expressed with gH/gL, the FRET signal from gB was significantly different from the construct containing CFP alone, as well as gB found in syncytia, indicating that this construct and others of similar design are likely to be powerful tools to monitor the conformation of gB in any model system accessible to light microscopy.

## Author Summary

Viral fusion proteins undergo complicated conformational changes in order to fuse viral and host membranes during viral entry. Conformational changes between prefusion and postfusion states also allow the virus to hide critical regions of the fusion machinery from the immune system. The structure of herpes simplex virus fusion protein gB is known only in its postfusion state, while the prefusion structure is unknown. To study the prefusion state, we created fluorescent protein (FP) insertions within gB and tested them for fusion activity. Due to the size of the fluorescent protein insertion, regions in gB that tolerate this insertion must be solvent exposed, thereby describing structural features of the prefusion structure. We created functional gB constructs with FP insertions in two of the three globular domains of gB, while non-functional insertions in the third domain suggested that it may be buried in the prefusion structure. Additionally, we created a dual-labeled FP gB construct which we found to report on the conformation of gB before and after fusion. Using this dual-labeled gB construct, we have demonstrated how fluorescence-based methods can be used to directly study dynamics of viral fusion proteins in living cells.

## Introduction

Herpes simplex virus infections are common, with severe disease striking some individuals while others are asymptomatic. Herpes simplex virus type 1 (HSV-1) afflicts roughly 70% of individuals within the United States [1], while HSV-2 afflicts approximately 16% [2]. Infection is chronic, with some individuals suffering only mild symptoms, while others experience frequent recurrence of viral lesions [3]. Ocular HSV infection can result in scarring of the eye, resulting in blindness if untreated [4]. Herpes encephalitis, most common among newborns, has a 20% mortality rate, although almost all survivors suffer neurological abnormalities [5]. Individuals with asymptomatic infection still shed significant amounts of virus, continuing to put others at risk [3].

HSV is an enveloped virus and enters a host cell by membrane fusion, either at the plasma membrane [6], [7], or through endocytosis [8] depending on the target cell type. Fusion requires the HSV fusion protein gB, and accessory proteins gH/gL and gD [9], [10]. gB and gH/gL are conserved throughout all herpes viruses, and these proteins have been described as the “core fusion machinery” [11]. Receptor binding protein gD is unique to most alphaherpesviruses, yet functionally equivalent proteins are found in the other two subfamilies of herpes viruses [11]. For HSV, gD binds a cellular receptor, such as Nectin-1 or HVEM, causing conformational changes that initiate a cascade of events leading to membrane fusion [12]. gD does so by activating regulatory protein gH/gL, which triggers gB to catalyze membrane fusion [13]. gB is the only essential HSV glycoprotein that must be membrane anchored in order to function, therefore confirming it as the viral fusogen [12], [14].

Viral fusion proteins such as HSV gB undergo large conformational changes to fuse host and viral membranes. Often regions of the structure essential for conformational change are buried in the protein fold, or obscured by glycan which can shield the protein structure from neutralizing antibodies [15], [16]. Knowledge of these conformational changes in other viruses have revealed mechanisms for certain neutralizing antibodies [17], [18], and have also led to therapeutics that target and inhibit viral entry [19], [20]. Conformational changes of viral fusion proteins consist of at least three states; a compact prefusion form, an extended intermediate form that spans both viral and host membranes, and a final postfusion form where the fusion protein has fused host and viral membranes by bringing together the fusion loops and transmembrane domain of the fusion protein [21]. Finer molecular detail has been difficult to resolve due to the topological constraints posed by viral and target membranes, as well as the heterogeneity of intermediate states. Nonetheless, an understanding of the fusion protein refolding process is tantamount to identifying vulnerable regions of fusion proteins for design of both vaccines and therapeutics.

**Figure 1:**
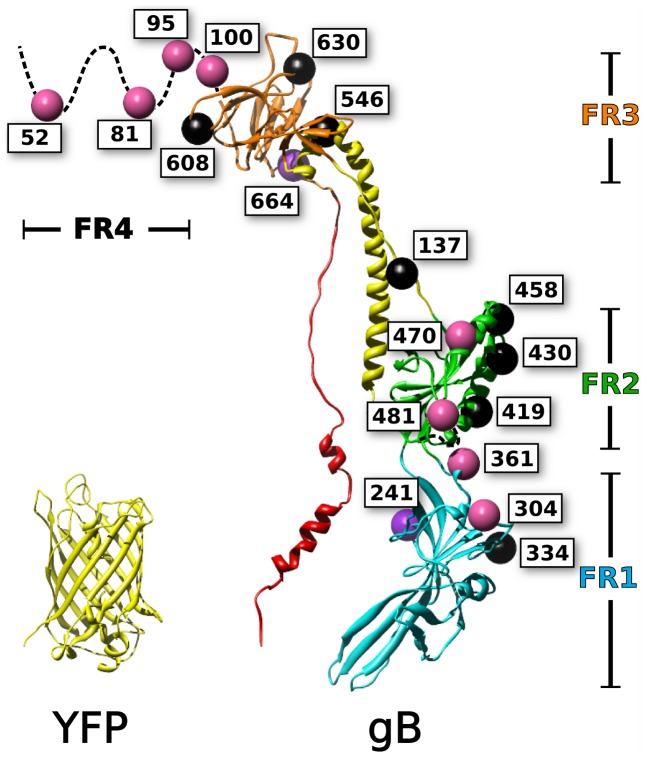
Mapping of fluorescent protein insertions on the gB crystal structure. Fluorescent protein insertions in HSV gB were selected from surface exposed locations on the postfusion crystal structure (PDB 3NW8 shown). The structure of YFP (PDB 1MYW) is shown to scale next to a protomer of the gB trimer. The four functional regions of gB are color coded by domain. Spheres marking the fluorescent protein insertion locations are colored to denote function: functional (pink), not surface expressed (black), surface expressed but non-functional (purple).

The structure of HSV gB [22] is conserved not only among other herpes viruses such as Epstein-Barr virus [23], but also across more distantly related viruses such as baculovirus gp64 [24] and vesicular stomatitis virus protein G [25], [26], all of which have been termed class III fusion proteins [27]. The crystal structure of HSV gB reveals a tall trimeric molecule with a long, helical central stalk (one protomer of which is shown in Fig. 1). [22]. The structure has been divided into four functional regions (FR) (Fig. 1), identified by four major immunogenic regions on HSV gB that are targets for virus-neutralizing antibodies [28]. FR1 is the first globular domain in the amino acid sequence, and contains the two fusion loops which form a fusion domain that mediates lipid binding by gB [29]. FR2 contains epitopes for neutralizing antibodies that block association with gH/gL, suggesting that important regulatory interactions with gH/gL are mediated by this region [14]. Following FR2 is an extended alpha helix that, together with the other subunits of the homotrimer, forms a central coiled-coil, also known as a central stalk. Trimerization of gB is mediated largely by contacts within the central stalk, which is a stable interaction as demonstrated by the thermostability of the trimer [30]. This extended central stalk brings together elements of the structure containing the fusion loops and the transmembrane domain, and thus is a hallmark of the postfusion state [19]. The C-terminus of the ectodomain packs against the central coiled-coil of the central stalk, and is believed to drive the fold-back that mediates membrane fusion [22]. Mutations in the C-terminus of the ectodomain have been shown to be hypofusogenic [31]. FR3 sits between components of the central stalk, and has been termed the “crown” due to its appearance in the crystal structure [22]. FR3 has also been described as a hinge due to its position in the sequence. The hinge action of the crown has been proposed to allow the fusion loops to extend towards the target cell and away from the transmembrane anchor in the viral envelope [22]. A membrane proximal region follows the C-terminus of the ectodomain and connects the ectodomain to the transmembrane anchor. The membrane proximal region has been shown to block the fusion loops from interacting with membrane [32]. The N-terminal 70 amino acids of the ectodomain, known as FR4, are not resolved in the crystal structure, likely because they are very flexible. The single example in the literature of a large insertion in the gB ectodomain is at position 42 (in the unstructured N-terminal region), where GFP was inserted without interfering with gB function [33]. This fluorescent protein construct, as well as others in the cytoplasmic tail of gB, facilitated study of trafficking and packaging of gB into the viral envelope [34].

Mutagenesis studies have been used to identify functionally important regions of gB, which are found throughout the ectodomain. Random linker-insertion mutagenesis, using linkers of five amino acids, revealed functional insertion constructs within the N-terminus, and within a loop in FR2 [35]. A separate study utilizing insertions of just two alanines described more sites that tolerated the smaller insertion, including sites in the N-terminus, FR1, and FR2 [36]. Still, the majority of the insertions in that study were functionally impaired; leading to the notion that gB was extraordinarily fragile to mutations.

Previous attempts at creating insertions in gB were done before knowledge of the gB crystal structure. We proposed that we could use the known structure of gB to create functional fluorescent protein (FP) constructs in the gB ectodomain. Because ongoing work to capture the gB prefusion structure has thus far been unsuccessful [30], we designed the FP insertion constructs with two goals: 1) to characterize structural features of the prefusion form of gB and 2) to create fluorescent tools for future studies. We created FP insertions at eighteen distinct sites in gB and found that eight were functional in fusion, some of which were temperature sensitive. Because of the size of the FP, we believe that these eight insertion sites are likely surface exposed in prefusion gB, and are also unlikely to be involved in critical interactions within the gB molecule or with regulatory proteins such as gH/gL. Functional insertion sites were located within two different functional regions of the gB ectodomain, as well as at multiple locations within the disordered N-terminus. To our knowledge, this is the first report of a fluorescent protein insertion within a core structural domain of any viral fusion protein. In working towards an assay to directly observe gB undergoing conformational change during fusion, we recombined two of our functional constructs to create gB with insertions of both CFP and YFP. We found this construct was functional, and produces a FRET signal when co-expressed with gH/gL. This construct and others like it provide a promising approach to understanding both the nature and timing of conformational changes in gB that drive membrane fusion.

## Results

### Fluorescent protein insertions were constructed throughout the HSV gB ectodomain

Based upon the postfusion crystal structure of HSV gB [37], sites were selected that were likely to accommodate fluorescent protein insertions. Residues were chosen based upon proximity to large regions of bulk solvent. Further consideration was given to the secondary structure of the selected sites by favoring loops and sequences that linked defined structural domains. We selected residues 81 and 470 based upon previous results obtained for linker insertion mutants that retained function [35]. Further mutations were selected across the surface of gB in an attempt to sample all structural regions that were exposed in the crystal structure (Fig. 1). The central helical stalk and C-terminus of the ectodomain were not targeted, because we reasoned that fluorescent protein insertions in these regions would sterically disrupt the known crystal structure. Each gB fluorescent protein insertion (gB-FP) was created by inserting an *Avr*II restriction site immediately following the residue index of the insertion's given name. The *Avr*II restriction site introduced a linker of 2 amino acids: Pro-Arg. Either Cerulean fluorescent protein (CFP), or Venus fluorescent protein (YFP), was ligated into the *Avr*II site, yielding a two amino acid linker of proline-arginine on either side of the fluorescent protein. For the CFP insertion construct at position 81 (gB-FP-81CFP), an additional glycine-serine linker was included (see methods). In total, 21 fluorescent protein insertion constructs were created, that corresponded to 18 unique sites (Fig. 1) and spanned the entire surface of the crystal structure, as well as the disordered N-terminus.

### Surface expressed gB-FP insertions are found in FR1, FR2 and FR4

Before assessing the function of gB-FP constructs, we first tested them for cell surface expression by CELISA, as this requires proper folding and trafficking of gB. Plasmids for each of the fluorescent gB constructs were transfected into B78H1 cells and incubated at either 37°C or 32°C for 24 h or 48 h, respectively. Cells were then fixed, and probed with a mixture of antibodies selected to target a diverse set of epitopes throughout gB. The antibody mixture included MAb A22 which binds FR3 [28], MAb SS55 which binds FR1 [28], MAb SS67 which binds FR3, and polyclonal IgG R68 which was raised against denatured gB.

**Figure 2:**
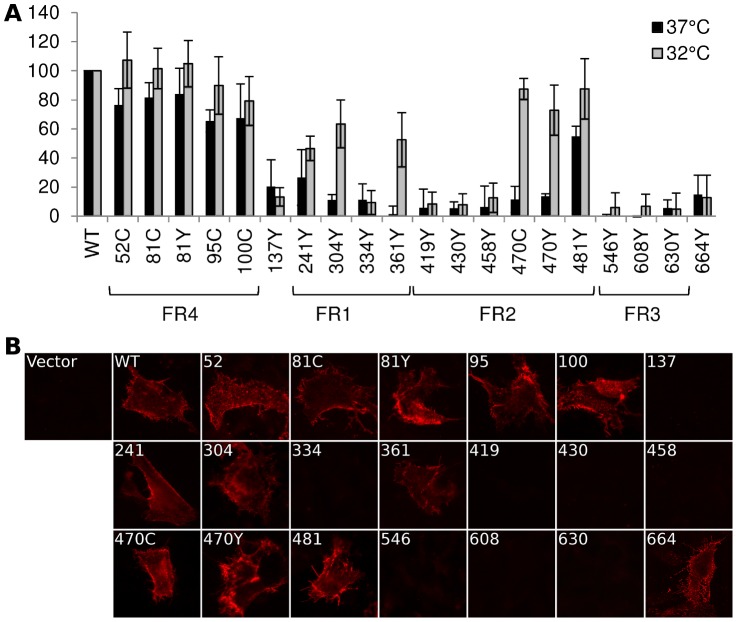
Cell surface expression by CELISA. (**A**) Cell surface expression of gB-FP constructs was probed by CELISA using a mixture of antibodies when protein was expressed at either 37°C (black) or 32°C (gray). Results are reported as a percentage of WT. (**B**) Immunofluorescence assay of gB-FP constructs expressed at 32°C. Monoclonal antibody A22 (red) was used to probe non-permeabilized cells, indicating cell surface expression.

When the cells were incubated at 37°C, all of the FR4 insertions exhibited substantial surface expression, as did the insertion at 481 in FR2 (Fig. 2). Insertions at position 52 and 81 (FR4) were expressed at approximately 80% of WT, while insertions 95 and 100 (also FR4) were expressed at approximately 65% of WT. Among the insertions located elsewhere in gB, only the insertion at amino acid 481 (FR2) had appreciable (60% of WT) surface expression. Therefore, at 37°C, only regions FR2 and FR4 tolerated FP insertions without seriously disrupting the global fold of gB.

As many of our FP constructs were not surface expressed at 37°C, we next considered if culturing cells at a lower temperature would improve surface expression. We repeated the cell surface expression assay, but incubated the cells at 32°C post-transfection and extended the growth time to account for the lower temperature. For constructs that showed some cell surface expression at 37°C, incubation at 32°C increased cell surface expression relative to WT (Fig. 2). Importantly, we also observed cell surface expression for five constructs that appeared null at 37°C (Fig. 2). Surface expression levels for a number of gB-FP constructs were indistinguishable from WT, including insertions at 51, 81, and 95. FP Insertions at positions 100, 470, and 481 were all approximately 80% of WT. gB-FP constructs 304 and 361 were both expressed at approximately 60% of WT, and 241 was expressed at 50% of WT. These last three insertions reside in FR1, which contains the fusion loops and is a critical domain for fusion [29]. Thus, a number of FP insertion constructs, particularly those in FR1 and FR2, were surface expressed predominantly at lower temperature.

We next used immunofluorescence assay (IFA) with non-permeabilized cells to visually confirm surface expression of all the gB-FP constructs. Since CELISA results indicated that all fluorescent gB constructs had improved cell surface expression compared to WT at 32°C, IFA was performed exclusively at 32°C. We used MAb A22, which binds gB with high specificity, to visualize gB on the cell surface. IFA results indicated that WT gB was expressed on the plasma membrane, while vector transfected cells were not bound by MAb A22 (Fig. 2B). In agreement with results from CELISA, gB-FP constructs 52, 81, 95, and 100 from the N-terminus were observed on the cell surface; as were FR1-localized gB-FP constructs 241, 304, and 361; and FR2-localized constructs 470 and 481. Additionally, we observed definitive surface expression for construct gB-FP-664 by IFA, although the number of these cells observed by IFA was small, likely explaining the low level of surface expression observed by CELISA. For FP insertions within FR3, a remaining concern was that these insertions might disrupt the A22 epitope in FR3 [28], leading to false conclusions about surface expression. To address this concern, we re-examined FR3 localized gB-FP constructs (546, 608 and 630) using rabbit polyclonal antibody R68, which was raised against full-length gB. R68 confirmed the conclusion based upon MAb A22 that these constructs were not surface expressed (Fig. S1).

### Functional gB-FP insertions were found in FR1, FR2 and FR4

**Figure 3:**
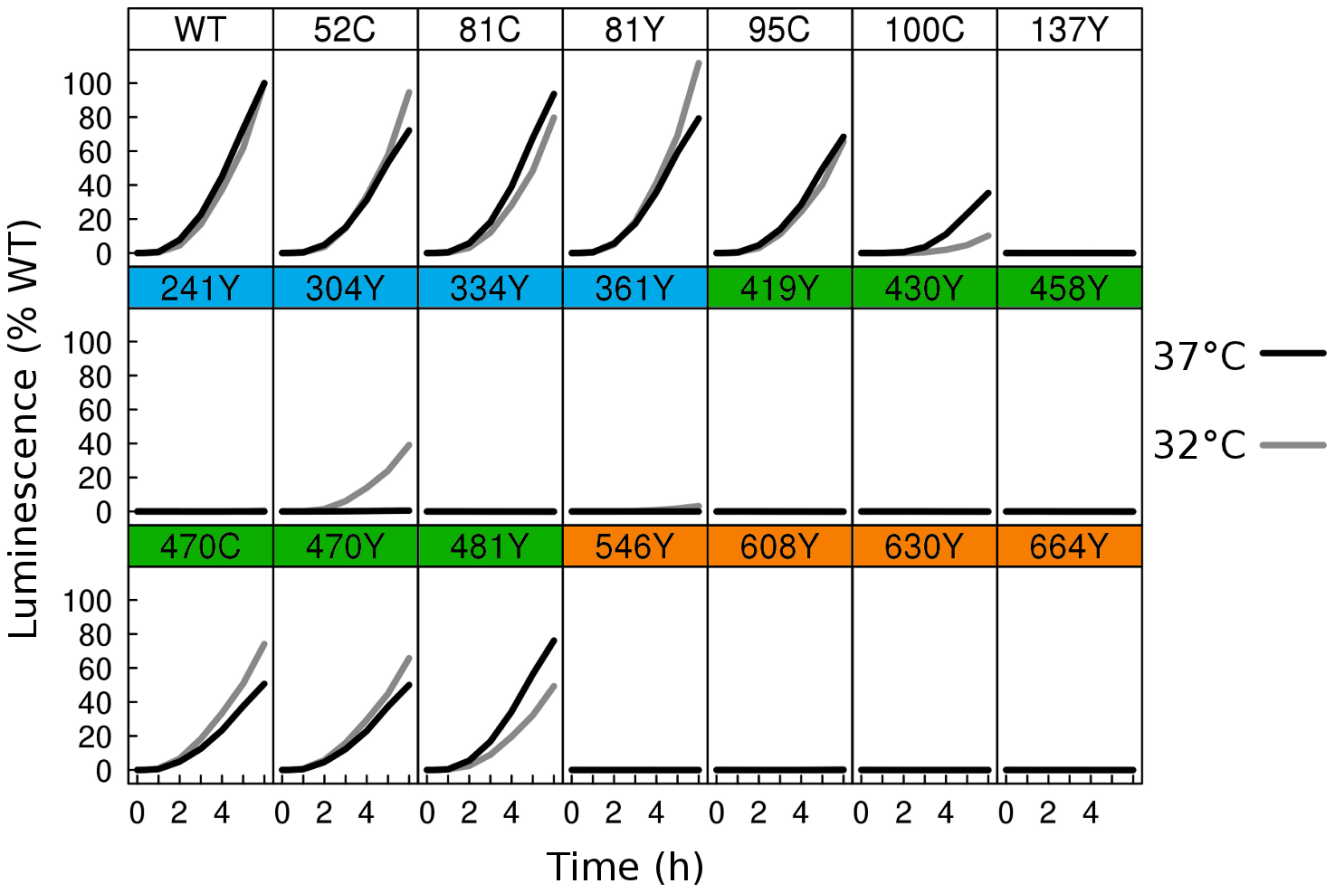
Fusion activity by dual split luciferase assay. Two distinct cell populations were prepared using receptor expressing cells and HSV glycoprotein expressing cells, each expressing half of a split Renilla luciferase reporter. HSV glycoproteins were expressed at either 37°C (black), or 32°C (gray). Using a membrane permeable Renilla luciferase substrate, fusion was monitored in live cells for 6 h from the time of mixing of the two cell populations. Luminescence values were normalized to the 6 h endpoint of WT. Activity of WT at 37°C was twofold higher than WT activity at 32°C.

We tested gB-FP constructs for activity using a cell-cell fusion assay based upon a split luciferase reporter [38]. Two cell populations were prepared, one expressing the HSV receptor Nectin-1 and transfected with a plasmid containing half of Renilla luciferase, and the other transfected with plasmids encoding HSV glycoproteins gD, gH/gL, and gB along with a plasmid for the other half of Renilla luciferase, as previously described [39]. When the two cell populations were mixed, cell-cell fusion was initiated, leading to the immediate reconstitution of luciferase. Using the cell permeable Renilla luciferase substrate EnduRen, fusion in living cells was monitored in real time. For cells cultured at 37°C for 24 h post-transfection, we found cell-cell fusion activity from 80–100% of WT for each of the FR4-localized gB-FP constructs (Fig. 3). Construct gB-FP-100, which lies closest to FR3, had activity 30% of WT, while none of the FR3 insertions had activity. gB-FP constructs 470 and 481 had activity of 50% and 70% of WT, respectively. Therefore these insertions allowed for gB function when expressed at 37°C, without disrupting either the global folding or function of HSV gB.

As many of the fluorescent protein insertions in gB had improved surface expression at 32°C relative to WT, we next asked if fusion activity for these constructs also improved at the lower temperature. We therefore repeated the split luciferase cell-cell fusion assay with cells cultured at 32°C post-transfection for 48 h, and we report the results as a percentage of WT activity at 32°C. Some constructs, but not all, had increased fusion activity at 32°C. gB-FP constructs in FR4 increased slightly in activity, approaching or slightly exceeding WT activity (Fig. 3). FR2 localized construct gB-FP-470 improved from 50% to 70%. Construct gB-FP-304 (FR1) was expressed on the cell surface only at 32°C, and gained fusion activity up to 40% of WT at this temperature. Construct gB-FP-304 is located in the same domain as the fusion loops; therefore fusion activity by gB-FP-304 was of particular interest. Fusion activity of gB-FP-361, also in FR1, was only 4% of WT at the 6 h endpoint, despite being expressed on the cell surface. While both gB-FP constructs 241 and 664 were surface expressed at 32°C as observed by IFA, neither of these constructs had fusion activity that could be distinguished from background. Lack of fusion by gB-FP-664 may be completely due to a low level of surface expression, since surface expression measured by CELISA for that construct was minimal. On the other hand, gB-FP-241 accumulated to 50% of WT on the cell surface, and lack of fusion activity likely represents a genuine functional impairment after reaching the cell surface. Constructs gB-FP-100 and gB-FP-481 decreased in fusion activity when the assay was done at 32°C as opposed to 37°C, suggesting that the rate limiting step in fusion for these two constructs was fundamentally different from the others, as they exhibited temperature dependence that was opposite from the others. In summary, by expressing the gB-FP proteins at 32°C, we observed fusion activity for gB-FP constructs in FR1, FR2, and FR4 of HSV gB, of which FR1 is notable for binding target cell membrane via the fusion loops.

**Figure 4:**
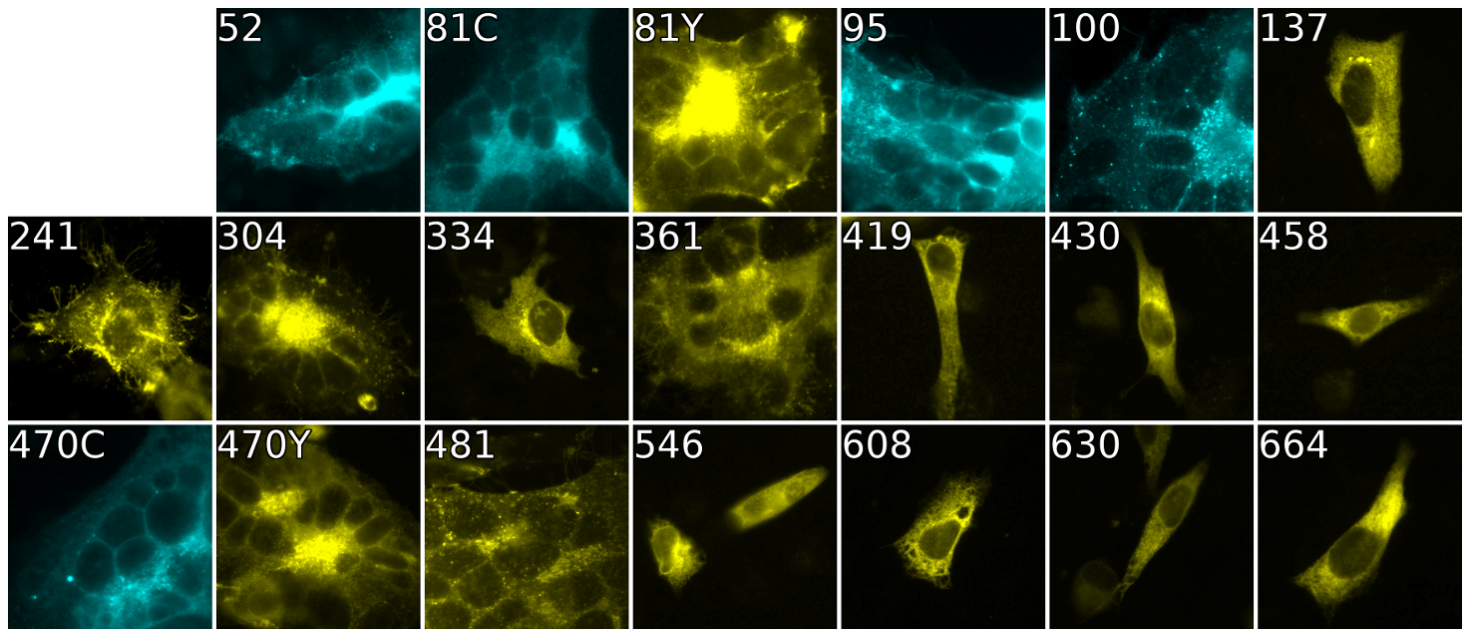
Cell-cell fusion with fluorescent gB constructs. Fluorescence of gB-FP constructs expressed in conjunction with gH/gL and gD were fixed after a 96 h expression interval at 32°C. All constructs exhibit fluorescence, but only gB-FP constructs 52, 81, 95, 100, 304, 361, 470, and 481 formed syncytia.

To confirm the luciferase assay results described above, we used fluorescence microscopy to visualize syncytium formation, a variant of the classic cell-cell fusion assay [40]. Cells were prepared that expressed Nectin-1 and the full complement of HSV fusion proteins: gD, gH/gL, and gB. After a 24 h growth at 37°C, cells were fixed and prepared for light microscopy. In agreement with split luciferase results, syncytia were observed for gB-FP constructs 52, 81, 95, 100, 470, and 481 (data not shown). Since lower temperature was permissive to surface expression for several gB-FP constructs, the assay was repeated at 32°C, and combined with an increase to 96 h of protein expression. Syncytia were readily observed for all constructs that were active at 37°C, plus insertions 304, and 361 (Fig. 4). The extended interval for protein expression is likely to have compensated for the slow rate of fusion for gB-FP constructs 304 and 361 that were observed in the split luciferase kinetic assay. At 96 h post-transfection we are observing the endpoint of cell-cell fusion at 32°C; as essentially no further activity was observed at a 120 h time point. This provides a comparison to the split luciferase assay performed 48 h post-transfection for 32°C expression, when protein was beginning to accumulate in measurable concentrations at the cell surface. Other constructs that were inactive in the split luciferase assay were confirmed to be inactive by light microscopy at this 96 h time point, including insertions 241 and 664 which were expressed on the cell surface at 32°C. Surface expression and cell-cell fusion activity for all gB-FP constructs is summarized in Table 1.

**Table 1:** Summary of constructs.

		Surface Expression (CELISA)		Surface Expression (IFA)	Fusion Activity (luciferase)		Fusion Activity (visual)
FR	FP Insertion	37°C	32°C	32°C	37°C	32°C	32°C
FR4	WT	+++	+++	yes	+++	+++	yes
	52-CFP	++	+++	yes	++	++	yes
	81-CFP	++	+++	yes	+++	+++	yes
	81-YFP	++	+++	yes	++	+++	yes
	95-CFP	++	+++	yes	++	++	yes
	100-CFP	++	++	yes	+	+	yes
FR1	137-YFP	+/−	+/−	no	−	−	no
	241-YFP	+	++	yes	−	−	no
	304-YFP	+/−	++	yes	−	−	yes
	334-YFP	+/−	+/−	no	−	−	no
	361-YFP	−	++	yes	−	−	yes
FR2	419-YFP	−	−	no	−	−	no
	430-YFP	−	−	no	−	−	no
	458-YFP	−	+	no	−	−	no
	470-CFP	+/−	+++	yes	+	++	yes
	470-YFP	+/−	+++	yes	+	++	yes
	481-YFP	++	+++	yes	++	++	yes
FR3	546-YFP	−	−	no	−	−	no
	608-YFP	−	−	no	−	−	no
	630-YFP	−	−	no	−	−	no
	664-YFP	+/−	+	yes	−	−	no
FR2+FR4	81-CFP-470-YFP	+	++	yes	+	++	yes

### A dual gB-FP construct, labeled in both FR2 and FR4, was surface expressed and functional

**Figure 5:**
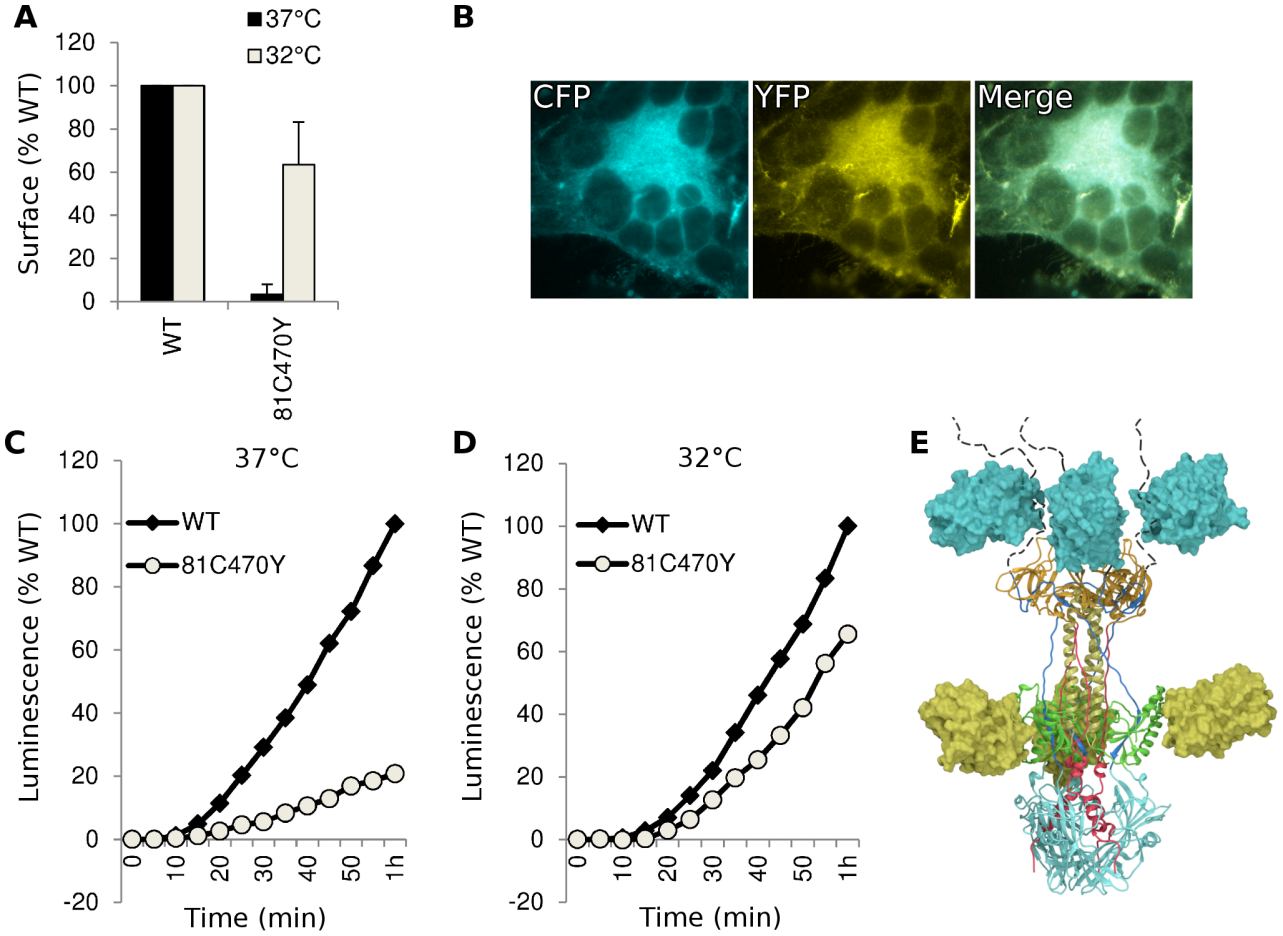
Characterization of dual-labeled construct gB-81C-470Y. (**A**) Cell surface expression by CELISA at 37°C (black bars) or 32°C (gray), normalized to WT levels. (**B**) Fluorescence images of cell-cell fusion with construct gB-81C-470Y at 32°C indicated that both CFP and YFP fluorescence was found in syncytia. (**C**) Fusion activity of gB-81C-470Y at 37°C and (**D**) fusion activity at 32°C. (**E**) Model of a gB trimer based upon the postfusion crystal structure, containing dual fluorescent protein insertions of CFP at position 81, and YFP at position 470.

Having found functional FP insertions in three different regions of gB, we asked if multiple fluorescent protein insertions could be combined into a single construct. We selected FP insertion constructs gB-81C and gB-470Y that were individually functional at 37°C. Insertions from these constructs were combined to create gB-81C-470Y, which contained two fluorescent proteins per protomer, or six fluorescent proteins per trimer. The fusion activity of this construct was approximately 20% of WT at 37°C, consistent with its poor surface expression (Fig. 5). When we expressed the dual-labeled construct at 32°C, we found both improved surface expression and an increase in activity to 60% of WT, suggesting that a limiting factor was protein folding and transport. We propose that protein folding improved at this lower temperature, thereby permitting an increase in trafficking to the cell surface and fusion activity. Thus the insertion of six FP molecules of the trimer did not disrupt the fusogenic activity of HSV gB. Here we have demonstrated that a single gB molecule can contain both fluorescent labels commonly employed in FRET studies (Fig. 5E), setting a precedent that dual-labeled fusion proteins can be generated that possess fusogenic activity.

### Antigenic characterization of dual-labeled gB-81C-470Y

An FP insertion occludes a region on the surface of gB that is similar in size to an antibody epitope, so we asked if the FP insertions blocked antibody binding to gB. We analyzed cell lysates from transfected cells using native western, and probed constructs gB-81C, gB-470Y, and the dual-labeled construct gB-81C-470Y with various antibodies whose epitopes are known in the postfusion conformation [28], [41]. The calculated molecular weight for monomeric gB is 97 kDa, but addition of glycans during trafficking increases the observed mass to approximately 110 kDa [42]. A fluorescent protein is 27 kDa, increasing the expected mass of gB-FP constructs to between 124 and 137 kDa. In the native western, two bands dominate for gB, a high molecular weight band which corresponds to gB trimer, and a lower molecular weight band which likely represents monomer.

**Figure 6:**
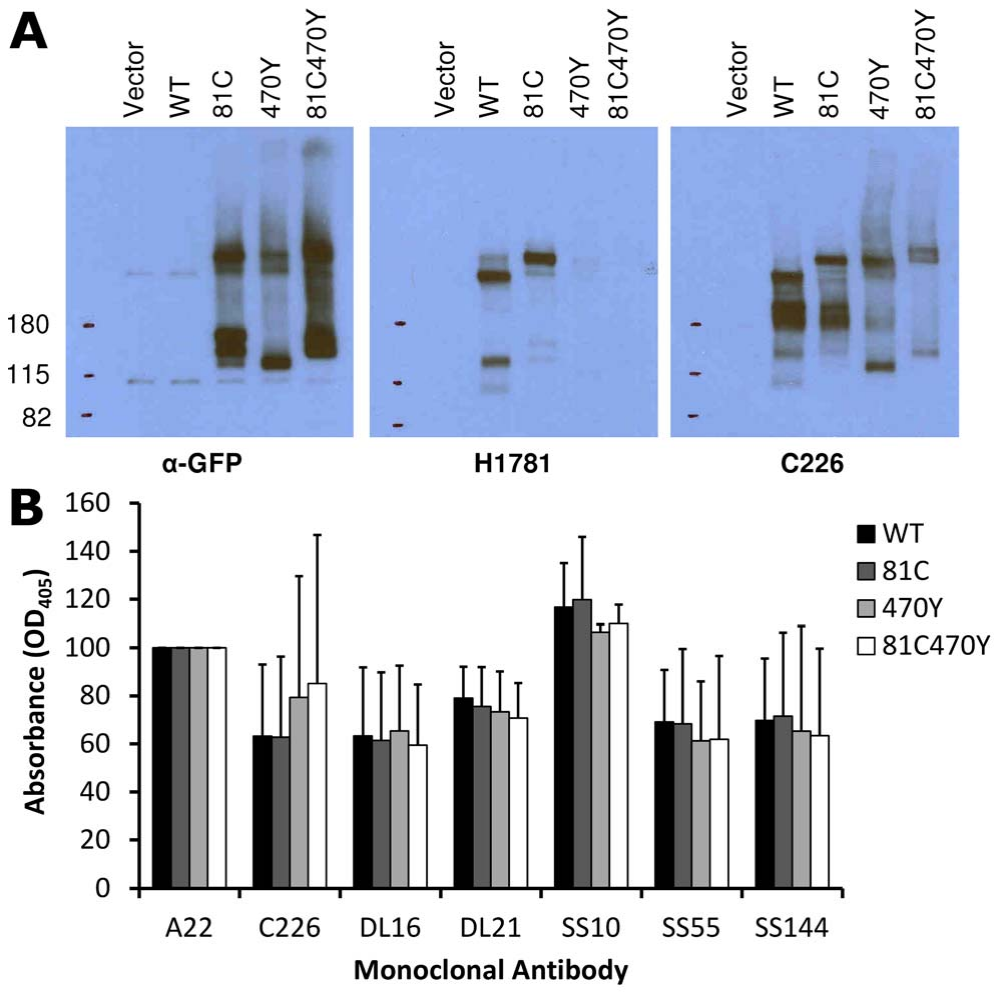
Antigenic analysis of insertion constructs at site 81 and 470. (**A**) Native western analysis of cell lysates from transfected cells expressing control or fluorescent insertions of gB, probed by three different antibodies. (**B**) ELISA was performed using polyclonal antibody to the gB cytoplasmic tail to capture gB-FP from cell lysates, and then probed for reactivity with a panel of monoclonal antibodies. Capture ELISA data was normalized to binding of WT gB to MAb A22. Bars represent the mean of three independent experiments, and error bars indicate the standard deviation.

First we probed the gB-FP constructs with an αGFP antibody. As expected, αGFP reacted with both monomeric and trimeric forms of each gB-FP construct, but neither with WT, nor with vector-only transfected cells (Fig. 6A). We next examined two antibodies with epitopes known to localize to FR2 and which were likely to be affected by the insertion at position 470. MAb H1781 binds a linear epitope at position 454–473 [43], and MAb C226 binds a continuous epitope that contains residue 414 [28], [41]. Both of these MAbs bind and neutralize virus. H1781 bound to both WT gB and gB- 81C, while H1781 did not react with either gB-470Y or gB-81C-470Y, indicating that the epitope for H1781 was either obscured or altered by the fluorescent protein at position 470. Next, we probed the three proteins with MAb C226 and found that, unlike H1781, C226 bound to constructs gB- 470Y and gB-81C-470Y. Therefore FR2 was only partially obscured by the fluorescent protein insertion at 470, while the epitope involved in C226 neutralization remained accessible.

We used a capture ELISA to determine the extent of similarity between WT and FP insertion constructs of gB. Polyclonal antibody specific for the cytoplasmic tail of gB was used to capture gB from cell lysates on an ELISA plate, and then the captured gB was probed with MAbs DL21 (conformational, competes with MAbs localized to the crown), SS55 (trimer specific, localized to FR1), DL16 (trimer specific, localized to FR1), SS10 (FR3 specific), and SS144 (FR1 specific) [28], [41]. Reactivity of MAbs with each of the FP insertion constructs and WT gB was similar, indicating that MAb epitopes were displayed on the FP insertion constructs equivalently to WT. This is consistent with each of the constructs adopting a similar tertiary structure, in spite of the FP insertions in 81C, 470Y, and the double insertion in 81C470Y (Fig. 6B). Included in this observation are MAbs that bind epitopes distant from the FP insertion sites, such as SS55 and SS144, as well as epitopes near the 470 insertion, such as C226. We conclude from the antigenic mapping that fluorescent protein insertions at these two sites did not broadly disrupt gB tertiary structure.

### FLIM-FRET measurements of gB-81C-470Y

Fluorescence resonance energy transfer (FRET) is a powerful technique that can detect if two fluorescent proteins are in close proximity to each other [44]. To establish if FRET can be measured with fluorescent gB constructs, we applied fluorescence lifetime imaging microscopy (FLIM) to our dual-labeled gB construct. Four different conditions were prepared for FRET analysis. We used the single Cerulean insertion construct gB-81C as a negative control, which accounts for Cerulean being both membrane anchored and present as a homotrimer through its linkage to gB. We compared gB-81C to dual-labeled Cerulean-Venus construct gB-81C-470Y expressed alone, or in conjunction with gH/gL. In a fourth condition, we induced cell fusion by adding soluble gD protein to cells transfected with gB-81C-470Y and gH/gL [14]. Proteins were expressed through transfection of B78H1-C10 cells (expressing Nectin-1) as described in surface expression and fluorescence assays, and live cells were imaged using time-correlated single photon counting, which measures the fluorescence decay at each pixel of an image. Each pixel is fit to an exponential decay, yielding the fluorescence lifetime. If the donor fluorophore, Cerulean in this case, is undergoing FRET with an acceptor fluorophore such as Venus, then the observed fluorescence lifetime of the donor fluorophore is decreased, and this commonly indicates that the distance between donor and acceptor fluorophores has decreased.

**Figure 7:**
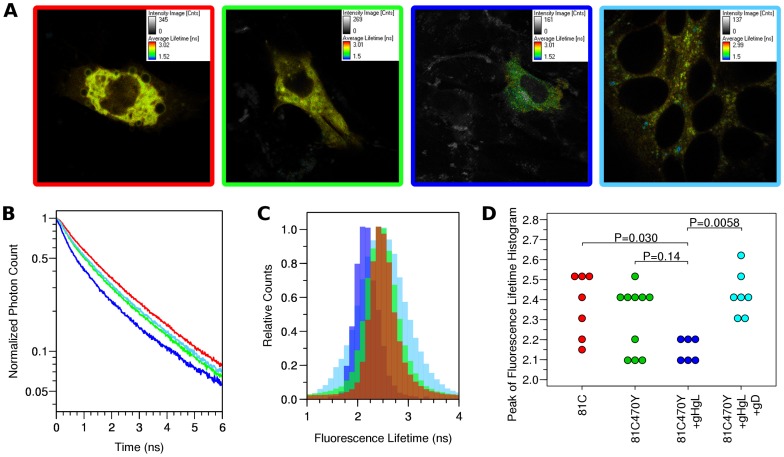
FLIM-FRET measurements. Cells were transfected with either gB-81C, gB-81C-470Y, gB-81C-470Y+gH/gL, or gB-81C-470Y+gH/gL+soluble gD, and color coded red, green, blue, and cyan, respectively. (**A**) Regions of interest were designated surrounding individual transfected cells, pseudo-colored by fluorescence lifetime. (**B**) Fluorescence decay curves are shown for cells chosen to represent the median fluorescence lifetime for the particular construct. (**C**) Donor fluorescence lifetime histograms corresponding to cells from *B*, where each histogram represents all the pixels of the FLIM measurement within the selected cell. (**D**) Taking the peak of the donor lifetime histogram for each cell as representative, these values were tabulated for all cells measured. Tukey's HSD test was used to determine P-values adjusted for all multiple comparisons between all three constructs.

Observed fluorescence decay curves describing individual cells did not fit a single exponential decay (Fig. 7), suggesting that more than one fluorescence lifetime was being observed within a single cell in all of the conditions analyzed. The distributions of fluorescence lifetimes observed for Cerulean within a cell are depicted in a fluorescence lifetime histogram (Fig. 7C, S2). It was not possible to identify compartments within the cell that showed distinct fluorescence lifetimes, so the regions of interest selected for the fluorescence decay curves and fluorescence lifetime histograms were chosen to represent the whole cell, and each cell was treated as an independent observation. Our efforts to decompose the fluorescence decay curves into the sum of two exponentials did not consistently result in two distinct lifetime values that had relevance to Cerulean, and was not pursued. A similar effort to fit the donor lifetime histogram to the sum of two Gaussian curves revealed that components with lifetimes much longer than expected for Cerulean were disrupting the curve fitting. A single Gaussian provided a reasonable fit to the peak of the donor lifetime histogram for most cells, while a few cells (one from gB-81C, one from gB-81C-470Y+gH/gL, and two from soluble gD) were clearly not described by this curve fit. Therefore we selected the peak of the donor lifetime histogram (the mode of the lifetime distribution) to represent the Cerulean fluorescence lifetime for the cell as a whole, and we found this was the best representation of the fluorescence lifetime for the most prevalent fraction of gB molecules within a cell (Fig. S2). Cells were ranked by their donor fluorescence lifetime, and cells that represented the median of the observed lifetimes for each construct were selected for Fig. 7 A–C. Fluorescence decay curves indicated modest differences, with the control having relatively slow decay (Fig. 7B). gB-81C-470Y initially had faster decay than the control, but at later time points within the decay curve, the slope of gB-81C-470Y was similar to the control, suggesting a subset of these proteins had shorter lifetimes, while another subset had lifetimes similar to gB-81C. The addition of gH/gL to gB-81C-470Y yielded the fastest decay curve at early time points, but also the largest curvature of the decay curve, indicating heterogeneity of fluorescence lifetimes within the cell, and therefore heterogeneity of either the gB conformation, or the local environment of Cerulean within the cell. When cell-cell fusion was induced by adding soluble gD to cells expressing gB-81C-470Y, gH/gL, and HSV receptor Nectin-1, the slope of the fluorescence decay curve was reduced. This suggested that even when selecting whole cells as our region of interest, we may be observing a net change in fluorescence lifetimes for the donor fluorophore Cerulean when converting gB from prefusion to postfusion.

Histograms of the Cerulean fluorescence lifetime reveal the distribution of fluorescence donor lifetimes within a cell. Comparing our four different conditions measured by FLIM-FRET, the peak of the fluorescence lifetime histograms were similar, except when gB was coexpressed with gH/gL, where the histogram peak was shifted to a shorter lifetime (Fig. 7C, S2). We calculated the mean and standard deviation the fluorescence lifetime histogram for all cells from each construct, which were 2.37±0.17 ns for gB-81C, 2.31±0.17 ns for gB-81C-470Y, 2.15±0.06 ns for gB81C-470Y co-expressed with gH/gL, and 2.43±0.11 ns for gB-81C-470Y co-expressed with gH/gL and supplemented with soluble gD to induce fusion. Given the number of cells that were observed, we needed a statistical test to evaluate if these differences were significant.

We used Tukey's HSD test in conjunction with an ANOVA to calculate multiplicity adjusted P-values describing the likelihood that each condition represented a different fluorescence lifetime distribution. Tukey's HSD test accounts for the multiple comparisons between each of the four conditions. We found that a P-value of 0.030 described the difference between the control and gB-81C-470Y expressed with gH/gL, while the difference between the control and gB-81C-470Y alone had a P-value of 0.14 (Fig. S3). Therefore, co-expression of gH/gL with gB-81C-470Y resulted in a statistically significant reduction in Cerulean lifetimes when compared to the gB -81C control, while gB-81C-470Y alone did not. The reduced fluorescence lifetimes indicated that positions 81 and 470 in prefusion gB are closer together when in the presence of gH/gL. This is direct evidence that gH/gL was influencing the conformation of gB, which, to our knowledge, has not been demonstrated previously. The effect of gH/gL on gB in the absence of gD was not anticipated, but this state most likely represents the prefusion conformation. Since gB-81C-470Y expressed alone was not significantly different from gB-81C, we are forced to conclude that gB-81C-470Y expressed alone must be in a “pre-prefusion” structure, as previous results expressing gB alone resulted in cell-cell fusion after gH/gL was provided *in trans* or as a soluble protein [12].

When we induced cell-cell fusion by adding soluble gD to cells that were co-transfected with gB-81C-470Y and gH/gL, fluorescence became more diffuse when spread across syncytia. Nonetheless, we applied the same analysis to these cells, finding that the mean lifetimes were statistically different from cells that did not have added gD, with an adjusted P-value of 0.0058 (Fig. 7D). We interpreted the increase in fluorescence lifetime of Cerulean found in syncytia to mean that the distance between Cerulean and Venus in the gB-81C-470Y construct increased as a result of cell-cell fusion. Therefore the change in FRET was a reporter for conformational change associated with fusion.

To ensure that the observed change in FRET was strictly due to interactions between Cerulean and Venus, we performed a negative control to determine if the fluorescence lifetime of gB-81C was affected by the coexpression of gH/gL, or by initiation of fusion with soluble gD. We repeated the FLIM-FRET analysis for these two conditions in addition to gB-81C alone, but we observed no change in fluorescence lifetime compared to gB-81C (Fig. S4, Fig. S5). While these new conditions were collected and analyzed independently, we found it informative to consider all the data at once, juxtaposing cells expressing gB-81C with those expressing gB-81C-470Y (Fig. S4E). If the same statistical analysis were performed across all of the FLIM-FRET data, we note that the two comparisons found to be statistically significant in our initial studies would remain so. An increase in the total number of statistical comparison without an increase in amount of data resulted in a slight increase in the P-value for comparisons between gB-81C-470Y coexpressed with gH/gL and and gB-81C-470Y with gH/gL and added soluble gD. The adjusted P-value decreased for the difference between gB-81C and gB-81C-470Y coexpressed with gH/gL, due to the additional data for gB-81C affirming the differences (Fig. S6).

## Discussion

Viral fusion proteins such as gB undergo large rearrangements of tertiary structure caused by conformational changes during viral fusion and entry. These structural changes are largely uncharacterized for HSV gB, as only the postfusion crystal structure is known. We designed fluorescent protein insertions in HSV gB to illustrate which gB domains will tolerate the steric bulk of an insertion of this size. If the insertion is functional, the result shows that the FP occupies a solvent exposed region on the surface of gB, i.e., one that is not buried by the tertiary arrangements of structural domains. Furthermore, the function of such a construct shows that this region is exposed at all steps of fusion, thus giving us information about structures for which there has yet to be a solution. Functional gB-FP constructs could also become tools for future characterization of gB structure through fluorescence-based methods.

We engineered fluorescent gB constructs by selecting regions from each domain of the HSV gB crystal structure that were conspicuously surface exposed in the postfusion structure. We anticipated that many of these gB-FP constructs would be non-functional, since the prefusion structure is likely to be very different from the postfusion structure. Furthermore, essential functions have been assigned to each of the structural domains of gB where we designed fluorescent protein insertions [14], [29], [43], [45], decreasing the likelihood of creating functional constructs. Indeed we found that many of these insertions disrupted proper surface expression of gB when expressed at 37°C. However, construct gB-FP-481, as well as several in FR4, were successfully surface expressed (Fig. 2), and were functional at 37°C (Fig. 3, 4). Furthermore, antigenic analysis of insertion constructs 81 and 470 indicated that the tertiary structure of these constructs or the dual-labeled construct was indistinguishable from WT (Fig. 6B). This result is an important extension of previous findings that these two regions tolerate short linker insertions [35], [36], or domain insertions at specific sites in either FR4 or FR2 [33], [46]. Even with small amounts of gB-470 on the cell surface (Fig. 2B), activity of this construct was still 50% of WT as measured by the luciferase cell-fusion assay (Fig. 3), indicating that fusion activity for this construct was greater than anticipated by surface expression levels. By expressing fluorescent gB constructs at a more permissive temperature of 32°C, we found that surface expression improved for gB-FP-470 in FR2, as well as for gB-FP-304 and gB-FP-361 in FR1. Large insertions in FR1 that retain function are unprecedented. FR1 is functionally critical to gB for at least two reasons: it includes epitopes that are targeted by neutralizing antibodies [28], and it contains the fusion loops which bind the target cell membrane [29]. FR1 is also sterically crowded, as postfusion gB molecules closely associate laterally on the membrane [47]. For these reasons, fusion activity by two fluorescent protein insertions located in FR1 should make them an important tool to dissect the mechanism of fusion by gB.

Insertion constructs located in FR4, the N-terminus of gB, were found to be surface expressed and functional, but not all N-terminal insertions had equivalent activity. gB-FP constructs 52, 81, and 95 all had robust fusion activity, although insertion 95 had less activity than the other two. Construct gB-FP-95 is separated by eight amino acids from FR3, and gB-FP-100 is just three amino acids away from FR3 in the crystal structure [37]. The fusion activity of gB-FP-100 was only 10% of WT rate in the 6 h fusion assay, yet fusion activity was readily observed at later time points (Fig. 4). Construct gB-FP-100 was one of only two constructs that had higher fusion activity at 37°C relative to WT than at 32°C relative to WT. For construct gB-FP-100 in particular, it is interesting to speculate that the added steric bulk of the fluorescent protein near FR3, which may serve as a hinge during fusion, could be impeding the transition between prefusion and postfusion conformations, and that higher temperature can overcome the steric encumbrance of the FP insertion.

In total, constructs from eight distinct insertion sites in gB were demonstrated to be functional when expressed at permissive temperature of 32°C, while constructs for eight distinct insertion sites were not expressed, and two sites were surface expressed, but non-functional (Fig. 1, Table 1). Functional gB-FP constructs inherently describe sites in gB that are not directly involved in critical interactions either within the gB molecule, across gB molecules, or with regulatory protein gH/gL. Significant constraints are placed upon a model of prefusion gB by considering both the connectivity of protein domains along with knowledge of which surfaces on these domains are solvent exposed, as determined by functional fluorescent protein insertions. Functional gB-FP constructs 304, 361, 470, and 481 broadly represent the outside surface of FR1 and FR2 in the postfusion structure, and our results indicate that these sites also make up the outside surface of these regions in the prefusion structure.

**Figure 8:**
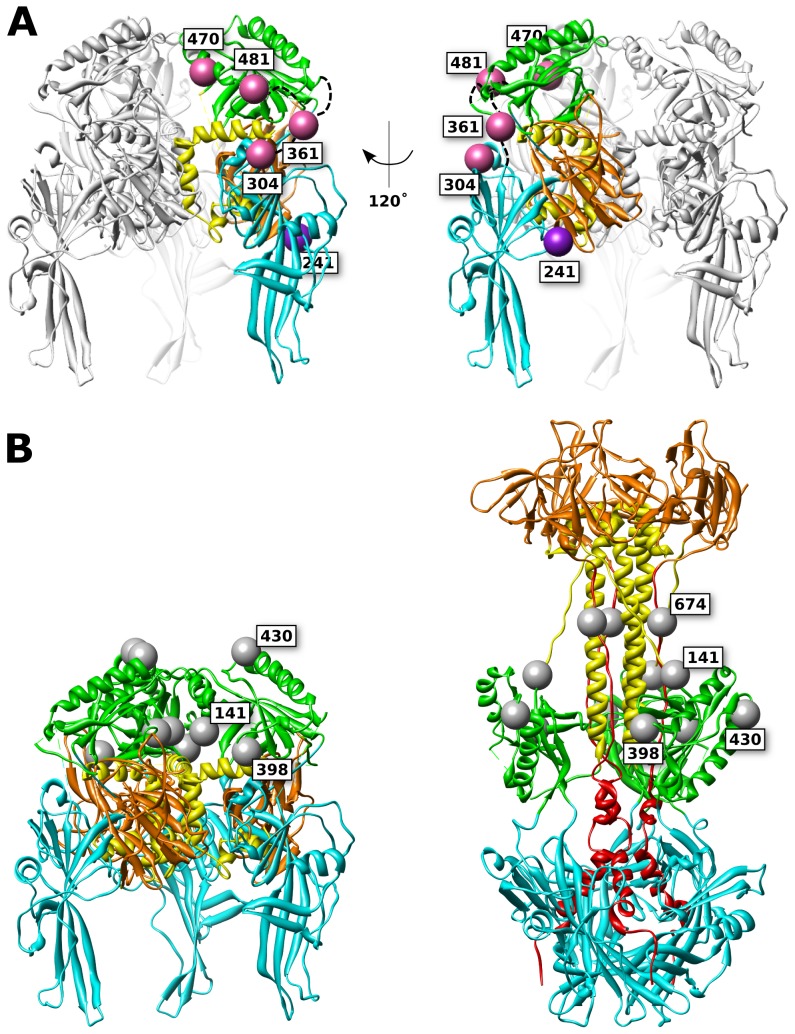
FP and glycan locations in a theoretical model of prefusion HSV gB. (**A**) Functional (pink) and surface expressed, non-functional (purple) FP insertions are mapped onto the prefusion model of HSV gB. The membrane is assumed to sit below the structure. Ribbon colors denote functional regions, as defined in figure 1. (**B**) Glycosylated residues are denoted in gray spheres. Glycosylation sites cluster at the top of the prefusion model (left), yet in the postfusion structure these sites are located at the girth of the gB structure (right), leaving the crown (FR3) exposed. Glycans that are found in resolved regions of the structure are positioned at 141, 398, 430, and 674. While position 398 may appear to lie at the center of the prefusion trimer, it is exposed to solvent from the side of the prefusion structure.

We have previously constructed a working model of the HSV gB prefusion structure based upon homology to vesicular stomatitis virus protein G (VSV G), as well as fusion rate measurements of HSV gB point mutants [39]. Here we consider the working model of the prefusion structure again in an effort to rationalize both functional and non-functional fluorescent protein insertions. The model (Fig. 8A, Supporting Data S1) was constructed with UCSF Chimera [48] by aligning domains from the HSV gB postfusion crystal structure [37] onto the prefusion crystal structure of VSV G [26]. The loops that link domains were not adjusted relative to the postfusion structure, therefore these loops often clash with neighboring domains. We argue that adjusting these loops without an appropriate template would merely obscure the shortcomings of the model while likely moving the model further from the true structure. The model was built on the assumption that tertiary structure rearrangements are the primary difference between prefusion and postfusion structures. By assessing the extent that experimental evidence supports this assumption for each of the domains, we would gain insight about the prefusion structure. The locations of functional FP insertions predicted by the prefusion model are consistent with the FP insertions facing outward towards bulk solvent in the model (Fig. 8A). The model is further supported by the prediction that the glycans, located in FR2, cluster at the top of the prefusion model, pointed away from the fusion loops and presumably outward from the viral envelope (Fig. 8B). Thus, they are predicted to be positioned on the outside of prefusion gB, shielding FR2 from neutralizing antibodies.

Non-functional FP insertion constructs that are surface expressed are an interesting case, because they suffer a defect in either regulation or execution of fusion. Examples of surface-expressed non-functional gB mutants have been rare, although two different insertions of dipeptides in FR2 were previously identified as such [36]. gB-FP constructs 241 and 664 (Fig. 1, purple spheres) were surface expressed at 32°C (Fig. 2), but did not function (Fig. 3, 4). Thus, these constructs could potentially either be trapped in the prefusion conformation, or emerge at the cell surface in the postfusion conformation which is incapable of fusion, and here we consider these two constructs separately.

Based upon our prefusion model of gB, amino acid 241 maps to a surface exposed face of FR1 adjacent to FR3 (Fig. 8). Positioned at the side of the prefusion structure, gB-FP-241 may block lateral interactions that are known to occur between gB trimers [47]. Alternatively, gB-FP-241 may block the approach of the regulatory protein gH/gL, which binds gB as an essential step for triggering fusion [12], [13]. As yet another possibility, construct gB-FP-241 could directly interfere with conformational changes between prefusion and postfusion structures. While we cannot conclude which mechanism causes gB-FP-241 to be fusion deficient, gB-FP-241 contains a fluorescent probe localized to a domain of gB critical to fusion regulation and activity, and may represent a trapped prefusion structure.

Insertion 664 resides in a linker between the crown and the C-terminal component of the central stalk. Very low levels of surface expression of gB-FP-664 were observed by CELISA (Fig. 2A); therefore lack of fusion activity by this construct may be due to inadequate amounts of gB-FP-664 that have accumulated on the cell surface. Still, we note that a minority of cells were observed by IFA to express gB-FP-664 in appreciable amounts (Fig. 2B), therefore we give additional consideration to the effect of insertion 664 on the structure. In the prefusion model, this site maps to a buried region of the crown, although the VSV G prefusion structure does not provide a template for the C-terminal component of the central stalk, and the direction of the amino acid chain following the crown, including position 664, is poorly predicted. The closest protein surface to insertion 664 is also shared by insertion 241 of a symmetry-related protomer, raising the possibility that these two insertions affect the same interface of gB. In the postfusion structure, site 664 is tucked under the crown near the central stalk, providing less room for the FP compared to the other insertion sites that were selected.

While the crown of gB (FR3) seems an attractive location for insertion mutations, being solvent exposed and well separated from other domains in the crystal structure, we found no functional gB-FP constructs that localized to the crown. We had previously suggested that FR3 is buried in the center of the prefusion structure [39]. If this is so, then it is not surprising that FP insertions in FR3 would cause steric clashes with FR1 and FR2. FR3 has also been previously reported to mediate binding to cell surfaces, in an apparent contradiction to FR3 being buried in the prefusion conformation [45]. This observation can be rationalized by noting that cell surface binding was measured with recombinantly expressed soluble gB, which is in the postfusion conformation. Therefore it has not been demonstrated that FR3 can mediate cell surface binding in the prefusion conformation. An alternative explanation is that the crown may interact with the cell surface at an intermediate step of fusion when gB is extended, after the fusion loops have engaged a target membrane, but when significant distance still separates viral and target membranes. Therefore it remains plausible that the crown forms the center of the prefusion structure, partially buried at its core. Our experimental results support this conclusion, as none of the FR3 insertions were surface expressed (Fig. 2), suggesting to us that they were unfolded and retained within the ER [49].

Fluorescence resonance energy transfer (FRET) measurements can provide a measure of distance between two fluorescent proteins, which is an attractive application of these gB-FP constructs. Dual-labeled, functional gB-FP constructs could be used to directly measure distance between gB domains either at the prefusion state, or in stable intermediates. Two major impediments have prevented FRET measurements in viral fusion proteins thus far. Fluorescent protein insertions have been viewed as too disruptive to viral fusion proteins that are already delicately balanced between prefusion, postfusion, and complete misfolding. With HSV gB, we have established several functional fluorescent gB constructs, and our success rate suggests that other interesting insertion sites remain to be found. Furthermore, we have created a dual-labeled fluorescent protein construct, indicating that individual insertions could be easily paired in a single construct. A second impediment to FRET studies of viral fusion proteins is caused by pH change, which is the trigger for conformation change in a number of well characterized viral fusion proteins, such as influenza hemagglutinin or VSV G [19]. Derivatives of either GFP or YFP are typically required for FRET measurements, and since GFP and YFP are quenched by low pH [50], quantitation measurement of FRET across a varying pH is intractable. HSV gB is capable of fusing at the cell surface [9], [51], and therefore circumvents the major problem of pH instability during endocytosis. Taken together, HSV gB-FP constructs present a surprisingly suitable model system to study viral fusion with real-time FRET observations.

We have performed preliminary FRET analysis using our gB-81C-470Y construct that contains both Cerulean and Venus. Based upon our prefusion model, we had predicted that the distance between insertion sites 81 and 470 would be shorter in the prefusion state than in the postfusion, but even if our prediction was correct, it was unclear that a difference could be observed by FRET. Using FLIM-FRET methodology, we found that there was a significant difference between our control construct gB-81C and gB-81C-470Y when the latter was co-expressed with gH/gL, indicating that FRET was occurring and the distance between sites 81 and 470 was reduced in this state (Fig. 7). We estimate an upper bound for this distance to be approximately 85 Å, based upon the Förster distance for Venus and Cerulean of 52 Å [52], the dependence of the FRET efficiency on the inverse sixth power of the distance between fluorophores [44], and assuming at least a 5% FRET efficiency was required for observation. Observed fluorescence lifetime values were similar between the gB-81C control and gB-81C-470Y when expressed alone, suggesting that for the majority of gB protein, FRET was not occurring when gH/gL was not present. Furthermore, the reduced Cerulean fluorescence lifetime was due to a FRET interaction with Venus, since the gB-81C construct expressed with gH/gL had similar fluorescence lifetimes to gB-81C alone (Fig. S4). When we added soluble gD to cells that were transfected with both gB-81C-470Y and gH/gL, cell-cell fusion commenced, and we observed longer fluorescence lifetimes again for Cerulean, similar to gB-81C when expressed alone. We interpret this to mean that gB transitioned from the prefusion to the postfusion state, which resulted in a loss of FRET signal. Thus, we infer that sites 81 and 470 are closer together in the prefusion state than in the postfusion (Fig. S7).

A surprising feature of the FLIM measurements was the overall short Cerulean fluorescence lifetimes, even in the control construct. The fluorescence lifetime of Cerulean alone has been measured as 3 ns, while Cerulean linked directly to Venus with a 5 amino acid linker has a lifetime of 1.6 ns [53]. The longest observed lifetime for our Cerulean alone construct, gB-81C, was only 2.6 ns, and the majority of data points had shorter lifetimes. Cerulean has previously been noted to undergo ‘energy migration’, or homo-FRET between proximal Cerulean molecules [54], which decreases the observed fluorescence lifetime. Two explanations are possible if this phenomenon occurs in gB-81C. If the N-terminus of gB closely associates with itself in the gB trimer, then three Cerulean molecules will be in close proximity, perhaps causing homo-FRET. A more mundane explanation may be that high level expression of a membrane protein was sufficient to saturate the local environment and drive homo-FRET. While we have examined the relative fluorescence lifetimes between our constructs, we note that to obtain definitive absolute measurements of fluorescence lifetimes, or to calculate FRET efficiencies that may be related to absolute distances between sites, it is critical that FLIM measurements be performed relative to a fluorescence lifetime standard, such as fluorescein [53], which we have not measured and is left for future work.

Our results illustrate how the FRET methodology can be used to assess the conformation of the viral fusogen gB in the context of living cells. The analysis could readily be extended in future work to make time dependent observations of gB conformation, perhaps synchronized by the addition of soluble gD as demonstrated here, or even soluble gH/gL [13]. Alternatively, viral particles could be directly studied, as demonstrated previously with fluorescently labeled capsid [55], although it remains to be demonstrated if gB-81C-470Y can be packaged in virus.

In summary, we have created fluorescent protein insertions in the HSV fusion protein gB, and interpreted the steric effects of these insertions, supporting a model of the prefusion state based upon VSV G. We found that gB functional regions 1 and 2 accommodate fluorescent protein insertions and therefore maintain a similar orientation to bulk solvent between prefusion and postfusion states. We have created a homology model of prefusion HSV gB, and found this model to be consistent with experimental observations of fluorescent gB constructs. We demonstrated that two functional fluorescent proteins could be combined within a single gB construct to create a dual-fluorescent gB molecule, while retaining function. We tested for FRET in our dual-labeled construct by observing Cerulean fluorescence lifetimes, and we obtained evidence of FRET when gB was coexpressed with gH/gL, while fluorescence lifetimes of gB in the absence of gH/gL was similar to the control. We added soluble gD to induce cell-cell fusion in cells expressing the dual-labeled gB construct, and found the FRET signal was again similar to the control, indicating that FRET was no longer occurring, and suggesting to us that the FRET signal was specific for a prefusion state. Finally, we demonstrated that the decreased Cerulean fluorescence lifetime in the presence of coexpressed gH/gL was strictly due to a FRET interaction with Venus by measuring FLIM-FRET for gB-81C coexpressed with gH/gL, finding that the fluorescence lifetime of gB-81C coexpression of gH/gL was not different from gB-81C alone. Using fluorescent protein insertions, we have leveraged experimental results to make concrete observations about the structure of prefusion gB, while creating novel tools to facilitate FRET observations of viral fusion protein conformational change during fusion.

## Materials and Methods

### Construction of fluorescent protein insertions in HSV gB

Mutations in gB were constructed from the HSV-1 KOS gB expression plasmid pPEP98 [56], since a crystal structure is available for this sequence [22]. Site-directed mutagenesis was performed with forward and reverse mutagenesis primers (Table S1), employing some protocol modifications to circumvent PCR problems caused by the GC-rich sequence of gB. Each 25 µl mutagenesis reaction was prepared with the following components: 0.5 µl Phusion DNA polymerase (New England Biolabs), 5 µl 5X GC Buffer, 0.5 µl 10 mM deoxyribonucleotides, 2 µl dimethyl sulfoxide (DMSO), 0.5 µl 10 µg/ml template plasmid, and 15 µl H_2_O. The reaction mix was split into 2 parts, and 0.5 µl of 10 µM primer (Forward or Reverse) was added to each. The PCR cycle began with 3 min at 96°C, followed by three repetitions of cycling between 96°C for 30 s and the annealing temperature for 15 s. The annealing temperature was selected using the nearest neighbor annealing temperature calculated by OligoCalc [57]. During this step, primers are extended to overcome primer-primer annealing [58]. Afterward, PCR was paused and forward and reverse primer reactions were mixed before commencing the standard site-directed mutagenesis procedure of denaturation, annealing, and extension. Product was *Dpn*I digested and ethanol-precipitated before re-suspending in H_2_0 and transformed into T10F' *E. coli* (Life Technologies) for DNA purification and sequence validation. Cloning was performed in the pUC19 cloning vector [59] modified to accommodate *Xho*I and *Bgl*II restrictions sites, which were used to shuttle validated sequences into the pCAGGS expression vector [60]. To facilitate potential recombination of gB constructs, a variant of pPEP98, named pJG1061, was created by site-directed mutagenesis containing silent mutations in gB introducing restrictions sites *Kpn*I at amino acid 266, *Hin*dIII at amino acid 544, and *Afl*II at amino acid 812 (Table S1). The function of this construct was identical to the parent construct (data not shown).

FP insertions were introduced by ligation into gB constructs that had been mutagenized to provide an *Avr*II restriction site, using the protocol described above. Plasmids were cleaved with *Avr*II, digested with shrimp alkaline phosphatase (New England Biolabs), and then ligated with fluorescent protein DNA fragments flanked by *Avr*II sites at both ends. Fluorescent protein DNA fragments with sticky ends were generated by PCR amplification and *Avr*II digestion from fragments of reference plasmid C32V, which contains Venus and Cerulean fluorescent proteins [53]. Fluorescent proteins were initially amplified with addition of an eight residue glycine-serine linker, which included the creation of CFP insertion construct (gB-FP-81CFP). Since all other fluorescent protein insertions made in parallel with gB-FP-81CFP containing glycine-serine linkers were non-functional, the glycine-serine linker was then omitted and all other gB-FP constructs were re-created without the linker, resulting in the constructs described in this work. A complete list of primers used for each construct is shown in Table S1. Individual clones were initially screened by PCR for correct orientation of the FP insert, followed by sequence confirmation of the construct. FP constructs are referred to by the number of the amino acid in of HSV-1 gB (KOS) that precedes the insertion (plasmid names given in parenthesis): 52-CFP (pJG1032), 81-CFP (pJG1024), 81-YFP (pJG1040), 95-CFP (pJG1048), 100-CFP (pJG1049), 137-YFP (pJG1050), 241-YFP (pJG1051), 304-YFP (pJG1052), 334-YFP (pJG1053), 361-YFP (pJG1054), 419-YFP (pJG1033), 430-YFP (pJG1034), 458-YFP (pJG1035), 470-CFP (pJG1038), 470-YFP (pJG1025), 481-YFP (pJG1055), 546-YFP (pJG1036), 608-YFP (pJG1056), 630-YFP (pJG1037), 664-YFP (pJG1057). A double FP insertion construct was created by utilizing the gB construct containing restriction sites introduced through silent mutations (pJG1061, see above). FP insertions from gB-81-CFP and gB-470-YFP were spliced together to create construct 81-CFP-470-YFP (pJG1026). FP insertions were visualized on the gB structure using the more recent PDB 3NW8 instead of the original gB structure PDB 2GUM because more residues of gB were resolved, including the sites of insertions 334 and 470 and the orientation of the N-terminus exiting the structure. Outside of the fusion loops, the domains in all of these structures have very similar conformations.

### Surface expression by CELISA

Cell-based ELISA (CELISA) was used to assess cell surface expression of gB as previously described [39]. Briefly, 2.5×10^4^ B78H1 cells [61] per well were plated on a 96 well plate and cultured overnight. The following day, cells were transfected using fugene6 according to the manufacturer's protocol (Promega). For each well, 0.6 ul fugene6 was combined with 50 ng empty vector DNA and 50 ng gB expression plasmid. Cells were incubated with transfection mix for 5 h at 37°C. Transfection mix was then removed, and fresh medium was added. For 37°C expression, cells were fixed after 24 h at 37°C. For 32°C expression, cells were fixed after 48 h. After formaldehyde fixation, cells were blocked with 5% non-fat dry milk in PBS with 0.2% tween 20, and then incubated for 1 h with 1 µg/ml primary antibody, which was a mix of polyclonal antibody R68 [45], and monoclonal antibodies A22, SS55 and SS67 [28], selected to capture gB in any antigenic state. Cells were rinsed three times with PBS, and then incubated for 1 h with a mixture of anti-rabbit and anti-mouse HRP conjugated secondary antibodies. Again, cells were rinsed three times with PBS, then 2,2′-azino-bis(3-ethylbenzothiazoline-6-sulphonic acid) (ABTS) substrate was added, and the optical density at 405 nm was recorded.

### Surface expression by IFA

Cell surface expression was visualized by immunofluorescence assay (IFA), as previously described [14]. 8×10^4^ B78H1 cells were plated on glass coverslips in a 24-well plate format and grown overnight. The following day, cells were transfected with fugene6 using 2.4 ul fugene6 and 400 ng total DNA per well (100 ng gB and 300 ng pUC19 vector carrier DNA). The transfection mix was removed after 5 h, fresh medium was added, and then cells were grown for 96 h at 32°C followed by formaldehyde fixation. Fixed cells were incubated 30 min in 50 mM NH_4_Cl, and then blocked 1 h in 10% goat serum. Cells were then incubated with 10 µg/ml A22 primary antibody in 10% goat serum for 3 h at room temperature (RT), rinsed three times, and then incubated with anti-mouse secondary antibody conjugated with Alexa-594 for 2 h at RT. Cells were rinsed three times with PBS and once with water, and then mounted with ProLong Gold (Invitrogen) anti-fade mounting medium, and photographed at 40× magnification using a C4742-95-12NRB camera (Hamamatsu).

### Activity by spit luciferase

Split Renilla luciferase constructs RLuc8_1–7_ and RLuc8_8–11_ [38] were used to quantitate fusion activity of fluorescent gB constructs. As described previously [39], two cell populations were prepared by transfection of B78H1 cells [61]. One cell population expressed HSV receptor Nectin-1, and RLuc8_8–11_. A second cell population expressed HSV glycoproteins gB, gD, gH, and gL from plasmids pPEP98, pPEP99, pPEP100, and pPEP101, respectively [56], as well as RLuc8_1–7_. For expression at 37°C, cells were assayed for 24 h post-transfection before activity was measured, and for expression at 32°C, cells were assayed after 48 h post-transfection. Fusion of cells expressing HSV receptor and glycoproteins resulted in reconstitution of the split Renilla luciferase protein, which was quantitated in live cells with membrane permeable luciferase substrate EnduRen (Promega).

### Activity by fluorescence microscopy

Direct observation of fusion activity was assessed using a cell-cell fusion assay to observe formation of syncytia using fluorescence microscopy. B78H1-C10 cells (expressing HSV receptor Nectin-1) [61] were plated on glass coverslips and transfected as described for IFA, except that each well of the 24 well plate was transfected with 100 ng DNA of each of the four HSV glycoproteins. For 37°C assays, cells were incubated overnight in transfection mix and fixed after 24 h. For 32°C assays, the transfection mix was removed after 5 h, fresh medium was added, and then cells were grown for 96 h at 32°C before formaldehyde fixation. Cover slips mounted and imaged as described above.

### Antigenic characterization by native western

293T cells were transfected using Gene Porter (Genlantis) with plasmid constructs expressing WT or mutant forms of gB. 48 h after transfection, cells were lysed for 1 h at 4°C in 50 mM Tris pH 7.5, 150 mM NaCl, 1% NP-40, and 1× complete protease inhibitor cocktail (Roche). Lysates were clarified by centrifugation for 30 min at 20,000× g and subjected to native SDS-PAGE and western blotting as described previously [62].

### Antigenic characterization by capture ELISA

To do capture ELISA, extracts of transfected cells were prepared as described above for native western blot analysis. Individual wells of a 96-well plate were first coated with 50 µl of 10 µg/ml polyclonal antibody R242 that was directed against the cytoplasmic tail of gB, for 2 h. Plates were then washed 3 times with PBS supplemented with 0.1% Tween-20 (PBS-T) and incubated in blocking buffer consisting of PBS-T supplemented with 5% non-fat dry milk. After 30 min, blocking buffer was removed and cell lysates, diluted 1∶10 in blocking buffer, were added for 1 h. Plates were then washed 3 times with PBS-T and incubated with 10 µg/ml MAb IgG in blocking buffer for 1 h. Plates were again washed 3 times with PBS-T and incubated for 30 min with goat anti-mouse horseradish peroxidase (HRP) diluted in blocking buffer. Finally, plates were washed 3 times with PBS-T, then ABTS substrate was added and optical density 405 nm was recorded. For each antibody, the signal generated from a control well containing no lysate was subtracted from the final reading.

### FLIM-FRET

Fluorescence lifetime imaging microscopy (FLIM) was applied to measure fluorescence resonance energy transfer (FRET) on fluorescent gB constructs. B78H1-C10 cells were plated on 35 mm dishes with glass cover slip bottoms (MatTek). Cells were transfected as described above, followed by a 3 day growth interval at 32°C. Soluble gD 306t [63] was added to indicated cells 4 h before imaging. FLIM measurements were made on live cells using a Leica TCS SP5 spectral imaging confocal/multiphoton microscope. Images were acquired using Cerulean excitation wavelength of 405 nm and the fluorescence emission was monitored at 452–491 nm, using a 60× objective with a 2.5× zoom. Time domain FLIM decay curves for Cerulean were recorded and fluorescence donor lifetime histograms were generated using SymPhoTime software (PicoQuant). Further analysis was performed using statistical software R (R Development Core Team). P-values adjusted for multiple comparisons were determined using ANOVA and Tukey's HSD test.

## Supporting Information

Data S1 **Coordinates of the working model for prefusion HSV gB.** Tertiary arrangement of HSV gB domains from postfusion gB crystal structure (PDB 3NWA) were aligned to the VSV G prefusion structure (PDB 2J6J). This PDB file specifies a single chain for the HSV gB prefusion model, but BIOMT tags permit the reconstruction of the trimer, such as with UCSF chimera's ‘biological unit’ tool.
(ZIP)

Figure S1 **IFA of non-permeabilized cells using R68 polyclonal antibody.** Since MAb A22 is known to bind the crown (FR3), fluorescent protein insertions in the crown region were screened for surface expression using R68 to validate A22 results. In agreement with A22 results shown in figure 2, none of the gB-FP constructs located in the crown were surface expressed.
(TIF)

Figure S2 **Donor lifetime histograms for all cells analyzed.** For each cell represented in figure 7, a histogram of the donor fluorescence lifetime depicts the distribution of lifetimes observed for each construct (**A**) gB-81C Cerulean control, (**B**) gB-81C-470Y, (**C**) gB-81C-470Y co-expressed with gH/gL, and (**D**) gB-81C-470Y co-expressed with gH/gL with added soluble gD (to induce fusion). The peak value of the histogram was chosen to represent the most prevalent fluorescence lifetime for each cell, and notated with a vertical dashed line on each histogram.
(TIF)

Figure S3 **Graphical representation of the 95% confidence interval for FRET measurements.** The distribution of donor lifetime measurements from all cells was assessed to determine if each of the constructs were significantly different from the others. Using the statistical software package R, an ANOVA was applied, followed by Tukey's HSD test. Shown in this figure is a graphical summary of the difference of means and the associated 95% confidence intervals for the comparisons. If the confidence interval does not span the origin in this plot (the difference of means is non-zero), then we conclude that the difference in the fluorescence lifetimes for the given pair of constructs is significant according to a 95% confidence interval. Significant differences are highlighted in gray.
(TIF)

Figure S4 **FLIM-FRET measurements of gB-81C with gH/gL and gD.** (**A**) Additional FLIM-FRET data were acquired for gB-81C expressed with gH/gL (magenta), gB-81C expressed with gH/gL and soluble gD protein (yellow), and gB-81C alone (red). Multiple cells were captured per image, and analysis was performed on the full set of cells per image to reduce the influence of cell-to-cell variability. (**B**) Fluorescence decay curves for the three conditions described in *A*. The image containing the median fluorescence lifetime for each construct was depicted. Curvature of the decay curves indicates multi-exponential decay, as seen with gB-81C-470Y. (**C**) Histogram of fluorescence lifetimes corresponding to decay curves in *B*, which largely overlap with each other. (**D**) Depiction of the peak of the fluorescence histogram for each image of each construct. Differences between the constructs were not significant according to Tukey's HSD test. (**E**) All data points from figure 7D were plotted in combination with additional data presented in *D*, juxtaposing the differences between gB-81C and gB-81C-470Y. Only in the presence of gH/gL was there a significant difference between gB-81C and gB-81C470Y.
(TIF)

Figure S5 **Donor lifetimes for gB-81C controls.** Fluorescence lifetime histograms, notated as described in figure S2, for (**A**) gB-81C, (**B**) gB-81C coexpressed with gH/gL, and (**C**) gB-81C coexpressed with gH/gL and with added soluble gD.
(TIF)

Figure S6 **Statistical analysis of all FLIM-FRET data.** All the data points for all constructs were combined as represented in figure S4E, and subjected to Tukey's HSD test. The 95% confidence interval is represented graphically, and instances where the confidence intervals for the difference in means are non-zero are taken as significant differences. Conditions that directly compare gB-81C and gB-81C-470Y are highlighted in gray. Of the three direct comparisons between gB-81C and gB-81C-470Y, significant differences were only observed when co-expressed with gH/gL. P-values corrected for multiple comparisons from Tukey's HSD test are listed for significant comparisons at the right of the plot.
(TIF)

Figure S7 **Interpretation of the FLIM-FRET data with respect to the prefusion model.** (**A**) The distance between CFP (Cerulean) and YFP (Venus) are predicted to be at or beyond the measurable distance for FRET in the postfusion structure. The crown of gB is separated by 70–80 Å from YFP at position 470, although 39 residues of unknown structure link CFP to the crown, causing uncertainty in the location of CFP. (**B**) As the first resolved residues of the prefusion model lie near the center of the structure in the crown, we suggest that the N-terminus may exit the prefusion structure either from the top or the side (N-terminus exiting through the side is shown), as the membrane would block the N-terminus from extending outward from the bottom of the model. Either route taken by the N-terminus would pass by YFP in FR2, therefore CFP and YFP are likely to be closer together in the prefusion state than they are in the postfusion state.
(TIF)

Table S1 **Primers used to generate gB fluorescent protein insertion constructs.**
(PDF)
