# Supplementary figures and images for: Functional Fluorescent Protein Insertions in Herpes Simplex Virus gB Report on gB Conformation before and after Execution of Membrane Fusion
Source: PLoS Pathog. 2014 Sep 18;10(9):e1004373. doi: 10.1371/journal.ppat.1004373 (PMC4169481; doi:10.1371/journal.ppat.1004373)

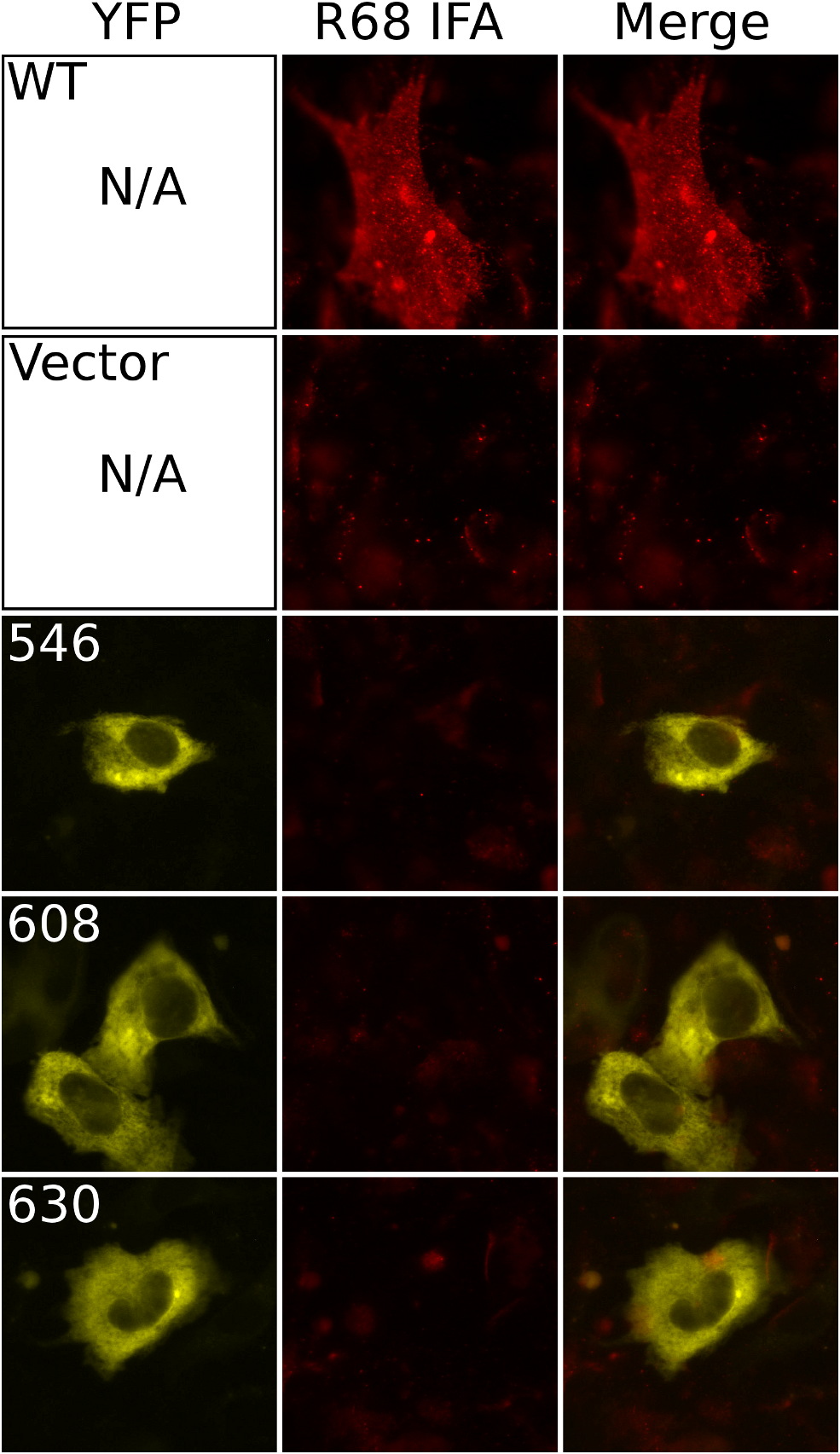

Supplement: Figure S1 — IFA of non-permeabilized cells using R68 polyclonal antibody. Since MAb A22 is known to bind the crown (FR3), fluorescent protein insertions in the crown region were screened for surface expression using R68 to validate A22 results. In agreement with A22 results shown in figure 2, none of the gB-FP constructs located in the crown were surface expressed. (TIF) [file ppat.1004373.s002.tif]

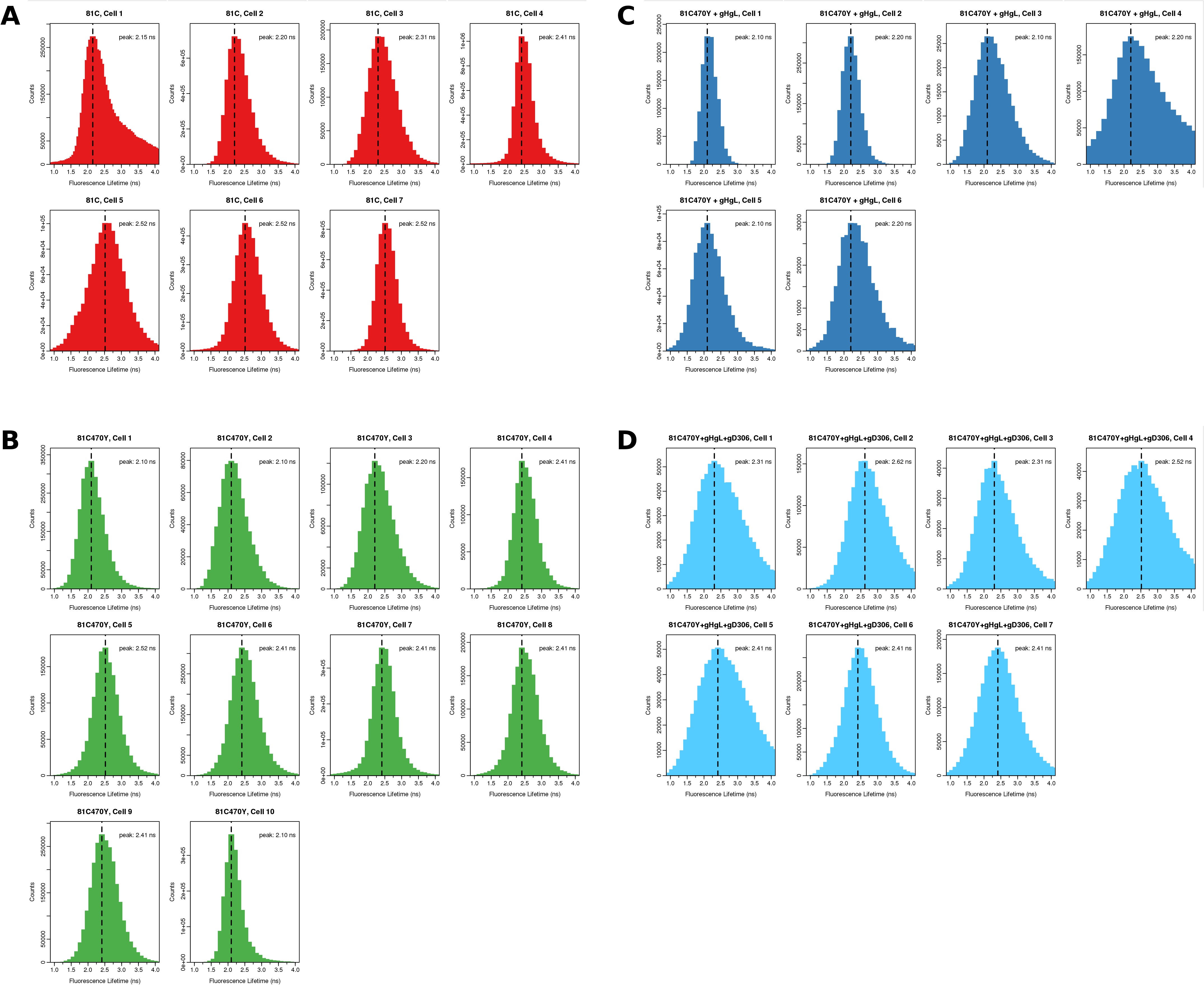

Supplement: Figure S2 — Donor lifetime histograms for all cells analyzed. For each cell represented in figure 7, a histogram of the donor fluorescence lifetime depicts the distribution of lifetimes observed for each construct (A) gB-81C Cerulean control, (B) gB-81C-470Y, (C) gB-81C-470Y co-expressed with gH/gL, and (D) gB-81C-470Y co-expressed with gH/gL with added soluble gD (to induce fusion). The peak value of the histogram was chosen to represent the most prevalent fluorescence lifetime for each cell, and notated with a vertical dashed line on each histogram. (TIF) [file ppat.1004373.s003.tif]

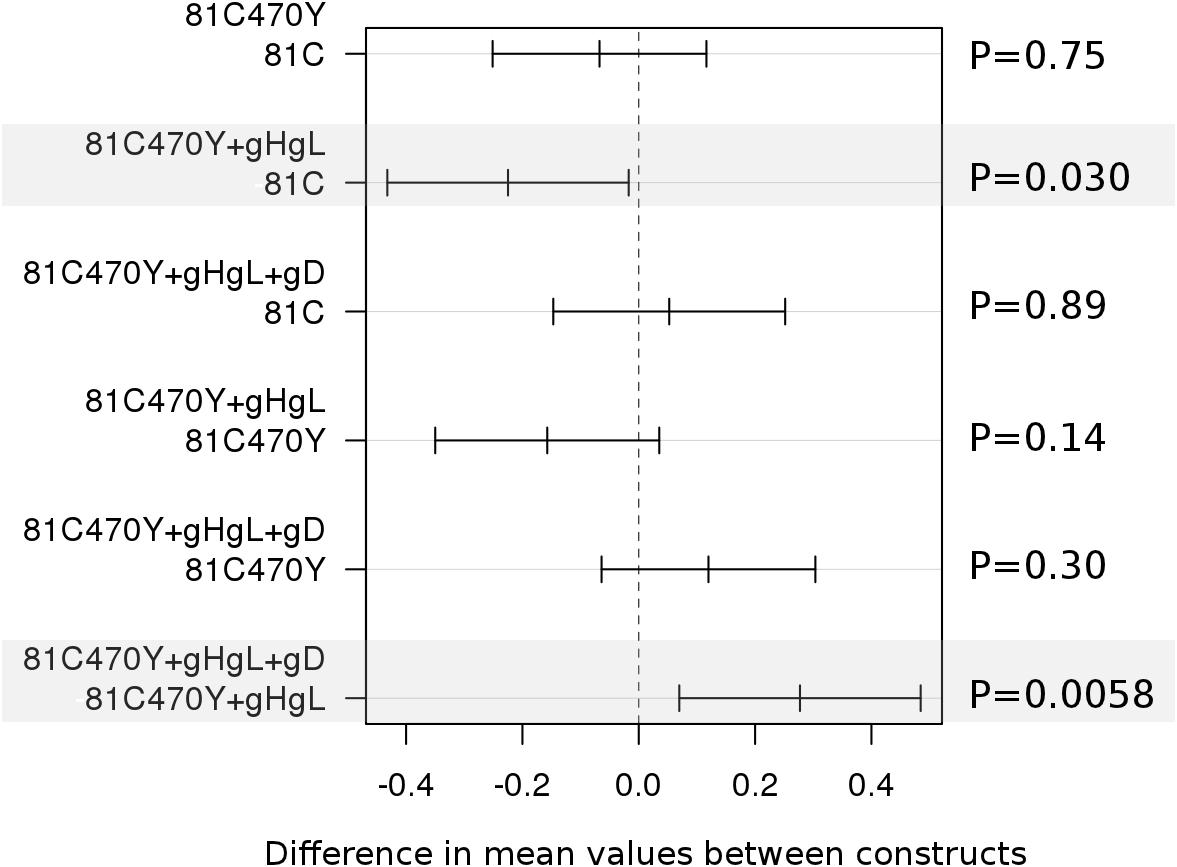

Supplement: Figure S3 — Graphical representation of the 95% confidence interval for FRET measurements. The distribution of donor lifetime measurements from all cells was assessed to determine if each of the constructs were significantly different from the others. Using the statistical software package R, an ANOVA was applied, followed by Tukey's HSD test. Shown in this figure is a graphical summary of the difference of means and the associated 95% confidence intervals for the comparisons. If the confidence interval does not span the origin in this plot (the difference of means is non-zero), then we conclude that the difference in the fluorescence lifetimes for the given pair of constructs is significant according to a 95% confidence interval. Significant differences are highlighted in gray. (TIF) [file ppat.1004373.s004.tif]

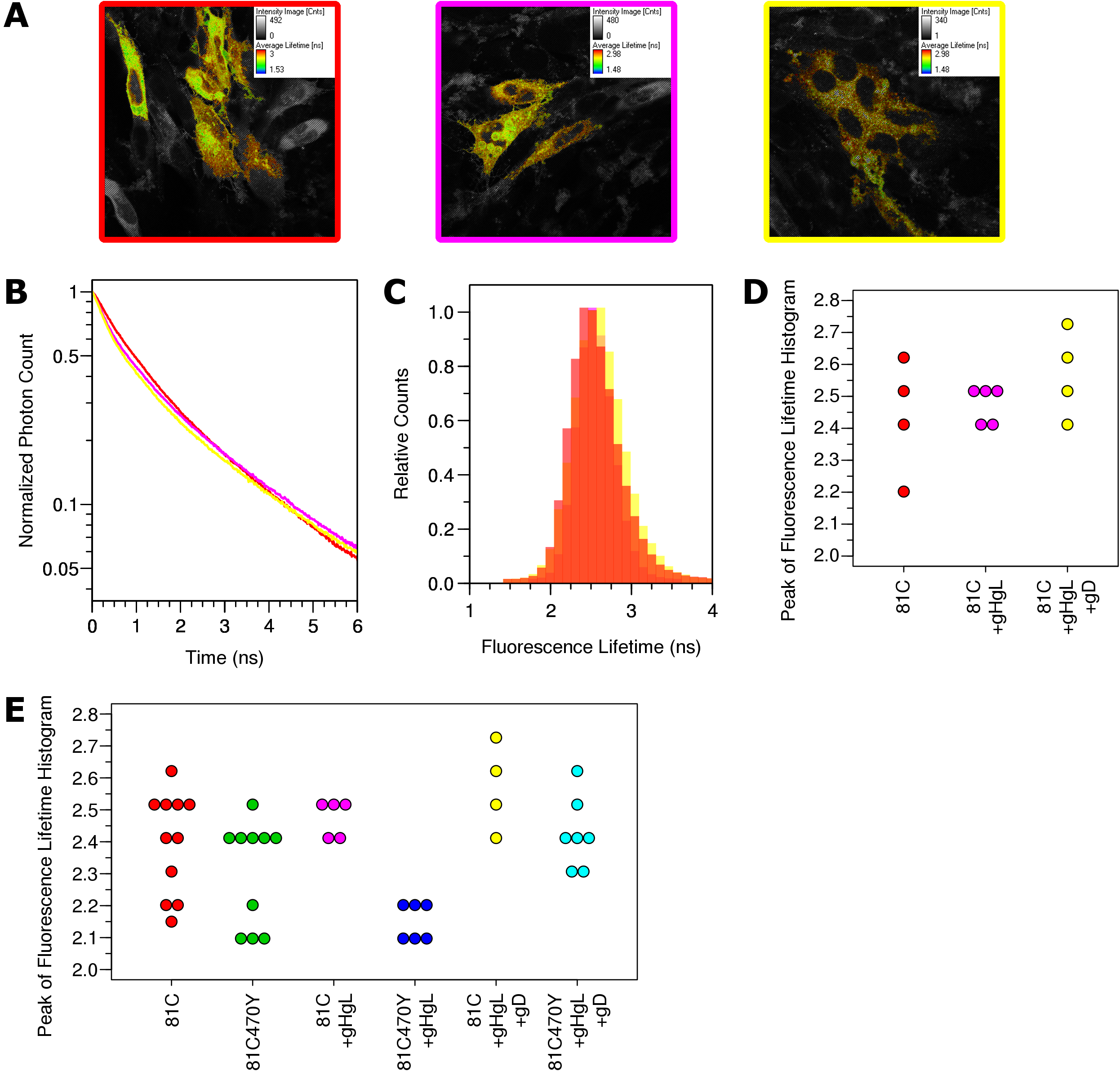

Supplement: Figure S4 — FLIM-FRET measurements of gB-81C with gH/gL and gD. (A) Additional FLIM-FRET data were acquired for gB-81C expressed with gH/gL (magenta), gB-81C expressed with gH/gL and soluble gD protein (yellow), and gB-81C alone (red). Multiple cells were captured per image, and analysis was performed on the full set of cells per image to reduce the influence of cell-to-cell variability. (B) Fluorescence decay curves for the three conditions described in A. The image containing the median fluorescence lifetime for each construct was depicted. Curvature of the decay curves indicates multi-exponential decay, as seen with gB-81C-470Y. (C) Histogram of fluorescence lifetimes corresponding to decay curves in B, which largely overlap with each other. (D) Depiction of the peak of the fluorescence histogram for each image of each construct. Differences between the constructs were not significant according to Tukey's HSD test. (E) All data points from figure 7D were plotted in combination with additional data presented in D, juxtaposing the differences between gB-81C and gB-81C-470Y. Only in the presence of gH/gL was there a significant difference between gB-81C and gB-81C470Y. (TIF) [file ppat.1004373.s005.tif]

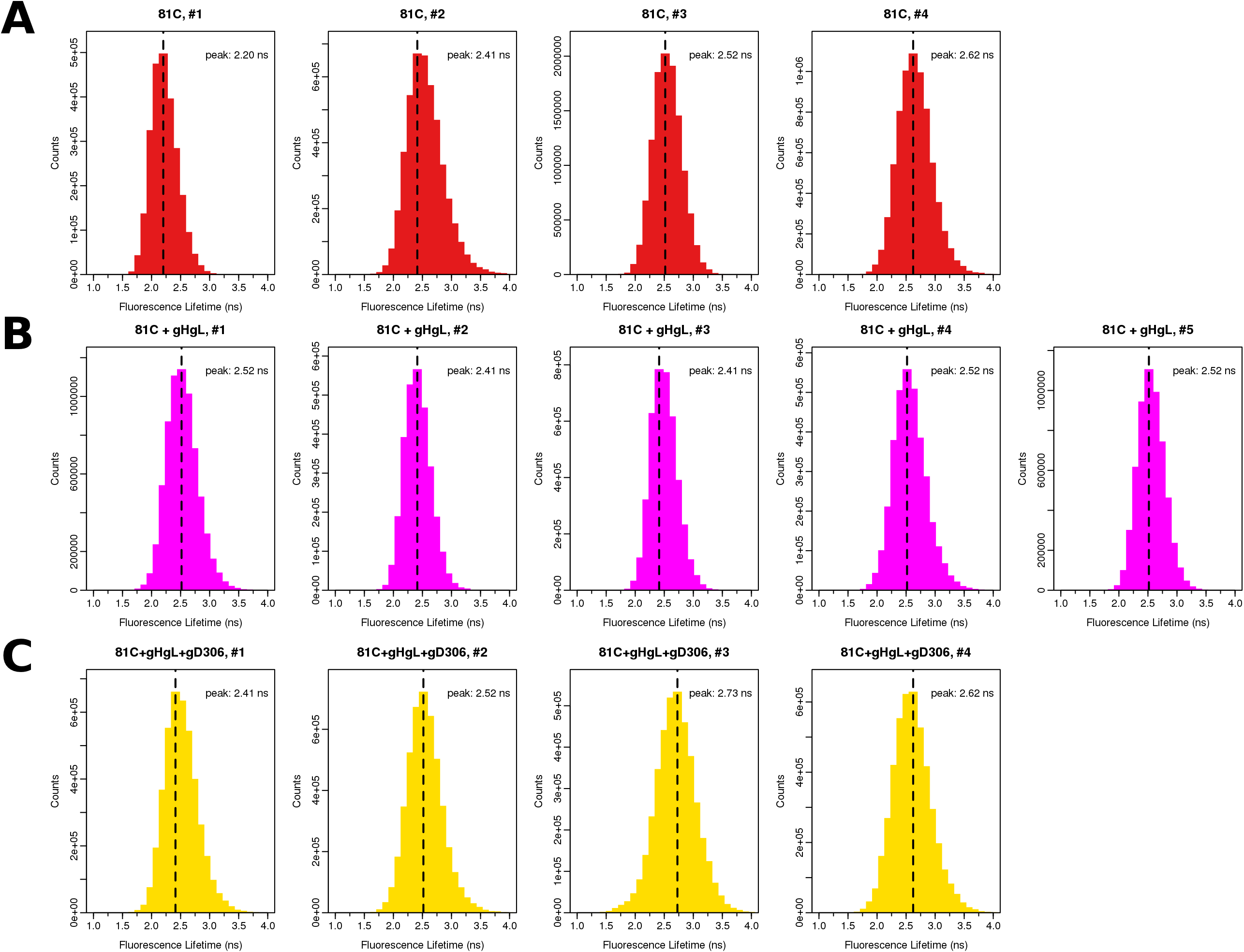

Supplement: Figure S5 — Donor lifetimes for gB-81C controls. Fluorescence lifetime histograms, notated as described in figure S2, for (A) gB-81C, (B) gB-81C coexpressed with gH/gL, and (C) gB-81C coexpressed with gH/gL and with added soluble gD. (TIF) [file ppat.1004373.s006.tif]

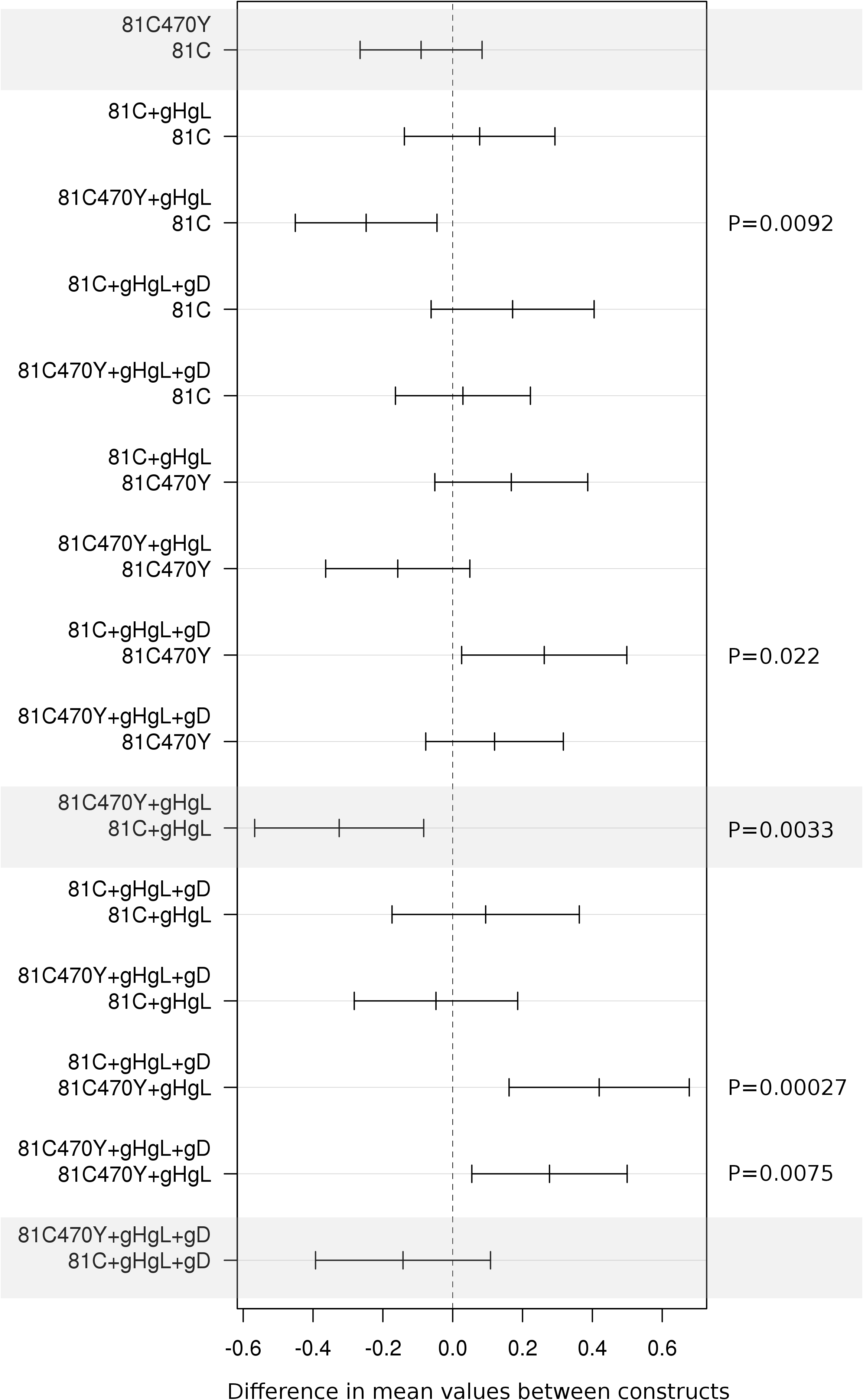

Supplement: Figure S6 — Statistical analysis of all FLIM-FRET data. All the data points for all constructs were combined as represented in figure S4E, and subjected to Tukey's HSD test. The 95% confidence interval is represented graphically, and instances where the confidence intervals for the difference in means are non-zero are taken as significant differences. Conditions that directly compare gB-81C and gB-81C-470Y are highlighted in gray. Of the three direct comparisons between gB-81C and gB-81C-470Y, significant differences were only observed when co-expressed with gH/gL. P-values corrected for multiple comparisons from Tukey's HSD test are listed for significant comparisons at the right of the plot. (TIF) [file ppat.1004373.s007.tif]

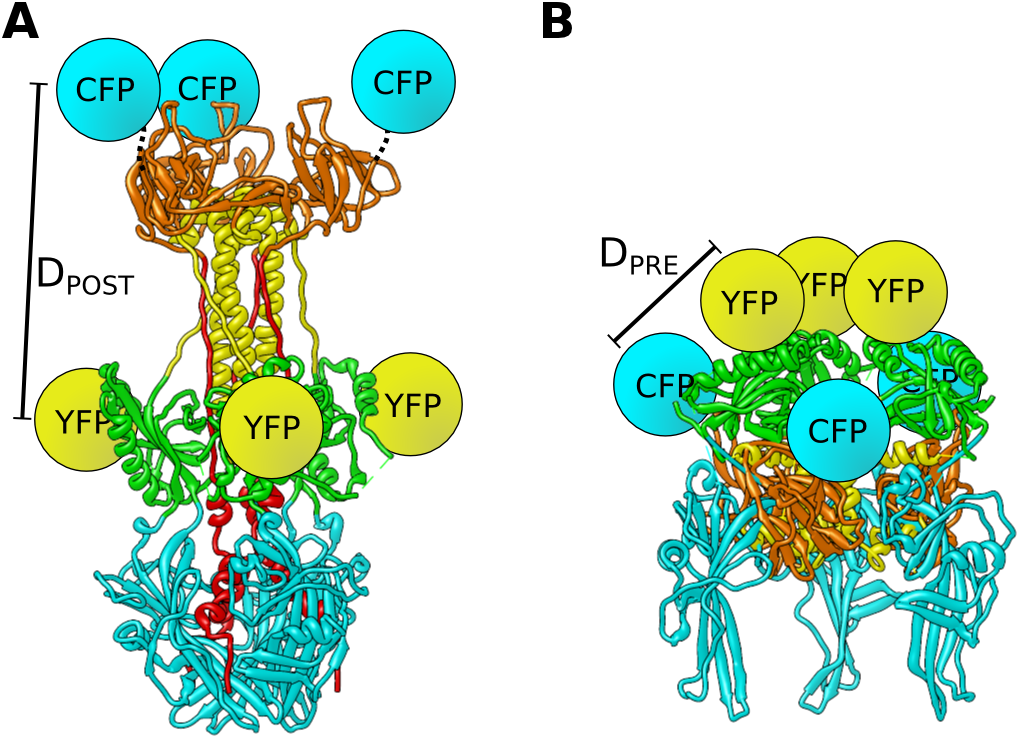

Supplement: Figure S7 — Interpretation of the FLIM-FRET data with respect to the prefusion model. (A) The distance between CFP (Cerulean) and YFP (Venus) are predicted to be at or beyond the measurable distance for FRET in the postfusion structure. The crown of gB is separated by 70–80 Å from YFP at position 470, although 39 residues of unknown structure link CFP to the crown, causing uncertainty in the location of CFP. (B) As the first resolved residues of the prefusion model lie near the center of the structure in the crown, we suggest that the N-terminus may exit the prefusion structure either from the top or the side (N-terminus exiting through the side is shown), as the membrane would block the N-terminus from extending outward from the bottom of the model. Either route taken by the N-terminus would pass by YFP in FR2, therefore CFP and YFP are likely to be closer together in the prefusion state than they are in the postfusion state. (TIF) [file ppat.1004373.s008.tif]
