# Supplementary material for: Functional Fluorescent Protein Insertions in Herpes Simplex Virus gB Report on gB Conformation before and after Execution of Membrane Fusion
Source: PLoS Pathog. 2014 Sep 18;10(9):e1004373. doi: 10.1371/journal.ppat.1004373 (PMC4169481; doi:10.1371/journal.ppat.1004373)
Supplement: Table S1 — Primers used to generate gB fluorescent protein insertion constructs. (PDF) [file ppat.1004373.s009.pdf]

Table S1. Primers used to generate gB fluorescent protein insertion constructs.

Application	Site[1]	Primer	Sequence[2]
Avr*II* insertion	52	Forward	AGGCGGCGAACGGGGGCCCTGCCCCTAGGACTCCGGCGCCGCCCGCCCTT
		Reverse	AAGGGCGGGCGGCGCCGGAGTCCTAGGGGCAGGGCCCCCGTTCGCCGCCT
	81	Forward	CAAAAAACCGAAAAACCCAACGCCACCACCTAGGCGCCCCGCCGGCGACAAC
		Reverse	GTTGTCGCCGGCGGGGCGCCTAGGTGGTGGCGTTGGGTTTTTCGGTTTTTTG
	95	Forward	ACCGTCGCCGCGGGCCACGCCCCTAGGACCCTGCGCGAGCACCTGCGGGA
		Reverse	TCCCGCAGGTGCTCGCGCAGGGTCCTAGGGGCGTGGCCCGCGGCGACGGT
	100	Forward	ACGCCACCCTGCGCGAGCACCCTAGGCTGCGGGACATCAAGGCGGAGAACA
		Reverse	TGTTCTCCGCCTTGATGTCCCGCAGCCTAGGGTGCTCGCGCAGGGTGGCGT
	137	Forward	CGCTGCCCGACCCGGCCCCCTAGGGAGGGTCAGAACTACACGGAGGGCATC
		Reverse	GATGCCCTCCGTGTAGTTCTGACCCTCCCTAGGGGGCCGGGTCGGGCAGCG
	241	Forward	CCGGCCAACGCCGCGACCCCTAGGCGCACGAGCCGGGGCTGG
		Reverse	CCAGCCCCGGCTCGTGCGCCTAGGGGTCGCGGCGTTGGCCGG
	304	Forward	CATGTCCCCGTTTTACGGCTACCGGCCTAGGGAGGGGTCGCACACCGAACACACC
		Reverse	GGTGTGTTCGGTGTGCGACCCCTCCCTAGGCCGGTAGCCGTAAAACGGGGACATG
	334	Forward	CGCGACCTCACCACCAAGGCCCCTAGGCGGGCCACGGCGCCGAC
		Reverse	GTCGGCGCCGTGGCCCGCCTAGGGGCCTTGGTGGTGAGGTCGCG
	361	Forward	GACTGGGTGCCAAAGCGCCCGCCTAGGTCGGTCTGCACCATGACCAAGTGGC
		Reverse	GCCACTTGGTCATGGTGCAGACCGACCTAGGCGGGCGCTTTGGCACCCAGTC
	419	Forward	ATCGGCAAGGACGCCCGCGACCCTAGGGCCATGGACCGCATCTTCGCCCGCA
		Reverse	TGCGGGCGAAGATGCGGTCCATGGCCCTAGGGTCGCGGGCGTCCTTGCCGAT
	430	Forward	ACCGCATCTTCGCCCGCAGGTACAACCCTAGGGCGACGCACATCAAGGTGGGCCA
		Reverse	TGGCCCACCTTGATGTGCGTCGCCCTAGGGTTGTACCTGCGGGCGAAGATGCGGT
	458	Forward	TCGCGTACCAGCCCCTTCTCAGCAACCCTAGGACGCTCGCGGAGCTGTACGT
		Reverse	ACGTACAGCTCCGCGAGCGTCCTAGGGTTGCTGAGAAGGGGCTGGTACGCGA
	470	Forward	CTGTACGTGCGGGAACACCTCCGACCTAGGGAGCAGAGCCGCAAGCCCCCAA
		Reverse	TTGGGGGCTTGCGGCTCTGCTCCCTAGGTCGGAGGTGTTCCCGCACGTACAG
	481	Forward	AAGCCCCCAAACCCCACGCCCCCTAGGCCGCCGCCCGGGGCCA
		Reverse	TGGCCCCGGGCGGCGGCCTAGGGGGCGTGGGGTTTGGGGGCTT
	546	Forward	TGGAACGAGGCCCGCAAGCTGAACCCTAGGCCCAACGCCATCGCCTCGGTCA
		Reverse	TGACCGAGGCGATGGCGTTGGGCCTAGGGTTCAGCTTGCGGGCCTCGTTCCA
	608	Forward	CCTGGTCAGCTTTCGGTACGAAGACCCTAGGCAGGGCCCGTTGGTCGAGGGG
		Reverse	CCCCTCGACCAACGGGCCCTGCCTAGGGTCTTCGTACCGAAAGCTGACCAGG
	630	Forward	TGCGGCTGACGCGCGATGCGATCCCTAGGGAGCCGTGCACCGTGGGACACCGGCGCT
		Reverse	AGCGCCGGTGTCCCACGGTGCACGGCTCCCTAGGGATCGCATCGCGCGTCAGCCGCA
	664	Forward	AGCTGAGCCGCGCCGACATCCCTAGGACCACCGTCAGCACCTTCATCGACCTCA
		Reverse	TGAGGTCGATGAAGGTGCTGACGGTGGTCCTAGGGATGTCGGCGCGGCTCAGCT
FP ampification	Linker	Forward	ATGCATCCTAGGGGCGGATCTGGCGGTGGATCTGGAGTGAGCAAGGGCGAGGAGCTG
		Reverse	ATGCATCCTAGGACCTGATCCTCCACCTGATCCTCCCTTATACAGCTCGTCCATGCCGAG
	No Linker	Forward	ATGCATCCTAGGGTGAGCAAGGGCGAGGAGCTG
		Reverse	ATGCATCCTAGGCTTATACAGCTCGTCCATGCCGAG
Kpn*I* mutation	266	Forward	TCCACCGGTACGGTACCACGGTAAACTGCATCGTCGAGGAG
		Reverse	CAGTTTACCGTGGTACCGTACCGGTGGAACGCCTCCACCC
Hind*III* mutation	544	Forward	GAGGCCCGCAAGCTTAACCCCAACGCCATCGCCTCG
		Reverse	CGTTGGGGTTAAGCTTGCGGGCCTCGTTCCACAGGG
Afl*II* mutation	812	Forward	CCACCAAGGAGCTTAAGAACCCCACCAACCCGGACGCG
		Reverse	TTGGTGGGGTTCTTAAGCTCCTTGGTGGTTAGAGGGTACAGGG

[1] Avr*II* site inserted following the specified amino acid number.

[2] insertion sequence underlined
